# Potential mammalian species for investigating the past connections between Amazonia and the Atlantic Forest

**DOI:** 10.1371/journal.pone.0250016

**Published:** 2021-04-09

**Authors:** Arielli Fabrício Machado, Camila Duarte Ritter, Cleuton Lima Miranda, Yennie Katarina Bredin, Maria João Ramos Pereira, Leandro Duarte

**Affiliations:** 1 Phylogenetic and Functional Ecology Lab (LEFF), Post-Graduation Programme in Ecology, Universidade Federal do Rio Grande do Sul (UFRGS), Porto Alegre, Rio Grande do Sul, Brazil; 2 Eukaryotic Microbiology, University of Duisburg-Essen, Essen, Germany; 3 Grupo Integrado de Aquicultura e Estudos Ambientais, Departamento de Zootecnia, Universidade Federal do Paraná, Curitiba, Paraná, Brazil; 4 Post-Graduation Program in Zoology, Museu Paraense Emílio Goeldi, Universidade Federal do Pará (UFPA), Belém, Pará, Brazil; 5 Faculty of Environmental Sciences and Natural Resource Management, Norwegian University of Life Sciences, Ås, Norway; 6 Bird and Mammal Evolution, Systematics and Ecology Lab (BiMa-Lab), Post-Graduation Programme in Animal Biology and Post-Graduation Programme in Ecology, Universidade Federal do Rio Grande do Sul (UFRGS), Porto Alegre, Rio Grande do Sul, Brazil; 7 Laboratório de Evolução e Genética Animal (LEGAL), Universidade Federal do Amazonas (UFAM), Manaus, Amazonas, Brazil; Universidad Austral de Chile, CHILE

## Abstract

Much evidence suggests that Amazonia and the Atlantic Forest were connected through at least three dispersion routes in the past: the Eastern route, the central route, and the Western route. However, few studies have assessed the use of these routes based on multiple species. Here we present a compilation of mammal species that potentially have dispersed between the two forest regions and which may serve to investigate these connections. We evaluate the present-day geographic distributions of mammals occurring in both Amazonia and the Atlantic Forest and the likely connective routes between these forests. We classified the species per habitat occupancy (strict forest specialists, species that prefer forest habitat, or generalists) and compiled the genetic data available for each species. We found 127 mammalian species presently occurring in both Amazonia and the Atlantic Forest for which, substantial genetic data was available. Hence, highlighting their potential for phylogeographic studies investigating the past connections between the two forests. Differently from what was previously proposed, the present-day geographic distribution of mammal species found in both Amazonia and the Atlantic Forest points to more species in the eastern portion of the dry diagonal (and adjoining forested habitats). The Central route was associated with the second most species. Although it remains to be seen how this present-day geography reflects the paleo dispersal routes, our results show the potential of using mammal species to investigate and bring new insights about the past connections between Amazonia and the Atlantic Forest.

## Introduction

Amazonia and the Atlantic Forest are among the most diverse tropical rainforests in the world [1, 2]. Biogeographical patterns of these South American megadiverse forests have been investigated since the 19^th^ century [3, 4]. Currently, the forests are separated by the ’dry diagonal’ comprising the Caatinga, the Cerrado and the Dry Chaco ecoregions. However, different sources of evidence, including biogeographical [5–10], palynological [11–13], and geological [14] show that these forests have been connected in the past.

The origin of the tropical rainforests in South America is dated to at least 65 million years ago (mya) [15–17]. Since then, these forests have undergone several changes, expanding and retracting. From the Oligocene (~ 23 mya) to the Pliocene (~ 3 mya), successive tectonic events led to the Andean uplift, restricting the entry of rainfall from the Pacific into the interior of the continent resulting in a drier climate with a forest reduction and the expansion of savannas, giving rise to the dry diagonal, and, consequently, the separation of Amazonia and the Atlantic Forest [15, 18–20]. Furthermore, there is evidence of more recent forest expansions and retractions caused by Quaternary climatic fluctuations, such as glacial cycles during the Pleistocene and recurrent periods of extreme rainfall during the Holocene [11–14]. Indeed, the high rates of vegetation cover changes in the dry diagonal and the Atlantic Forest [21, 22] suggest not just ancient but also recent connections and disruptions between Amazonia and the Atlantic Forest [9].

Although the time scale of changes in South American rainforests can be extremely large, making it difficult to accurately punctuate the total possible connective routes between these forests, three routes have been suggested: one through the forests of North-eastern Brazil (the northeast route), another through the gallery forest of the Brazilian Cerrado ecoregion (the central route) and a third through the forests of the Paraná Basin, the Moist Chaco, and the Pantanal (the southeast-northwest route) [5, 6, 23]. To facilitate the nomenclature, we will hereafter refer to these routs as the Eastern, Central, and Western routes.

According to Por [5], the Western route would have been the most ancient connection and would have occurred more often over time, followed by the Eastern route. In a study aimed to test Por’s hypothesis [5], Ledo & Coli [10] reviewed the literature for molecular evidence of connections between Amazonia and the Atlantic Forest for ca. 60 vertebrates, including 10 mammals. They found more studies that evidenced connections through the Western route (with most evidence dating from the Miocene) compared to the Eastern route (dated to the Pleistocene) [10]. However, this result could be biased due to the poor sampling in the north-eastern region [24]. Thus, it remains uncertain whether the Western route was indeed the most frequently formed connection and therefore the most used route in the past.

Furthermore, although three routes have been proposed, some authors considered only two major routes. For example, both Batalha-Filho et al. [8] and Ledo & Colli [10], did not consider the Central route as an independent route but included it as part of the Eastern route. However, the Central route has been well documented as a potential separate migratory pathway in the literature for both animals and plants [5, 6, 23, 25, 26]. Moreover, investigating the phylogeography of eight small mammals, Costa [6] found a larger number of related, small mammals occurring in Amazonia and the Atlantic Forest that could have come through the Central route. However, this study was limited to small mammals which have specific traits, such as limited dispersion ability. Considering more species with different traits and divergence times may therefore add further evidence on the past use of the Central connection, as well as for the other routes, and their time scales.

Other molecular studies investigate the role of the historical connections between Amazonia and the Atlantic Forest in terms of dispersion and diversification of several animal species, such as mammals [6, 27], birds [8], reptiles [28–30], amphibians [31] (for a literature revision of vertebrate evidence see [10]), and insects [32]. Yet, the totality of species that may evidence past connections between Amazonia and the Atlantic Forest has not been mapped and such information is particularly scarce for mammals. In this context, a compilation of available data, including geographic, ecological, and genetic data, for species that could be used to test the past connections between Amazonia and the Atlantic Forest could assist future investigations of the aforementioned hypotheses.

Here we aim to identify mammal species of potential use for investigating the past connections between Amazonia and the Atlantic Forest through geographical distribution patterns, habitat preferences, and genetic data. For this, we created a list of mammal species that occur in both Amazonia and the Atlantic Forest and we identify their possible past use of the three proposed connective routes. We highlight the potential of using these species to further investigate the frequency and time scales of the connective routes. We believe that our results may serve as a basis for future biogeographic studies considering different mammalian taxa to test hypothesis about connection routes between Amazonian and Atlantic forests.

## Material and methods

We considered mammal species to be of interest for investigating the past connections between Amazonia and the Atlantic Forest if they fulfilled the following two criteria: they had to 1) occur in both Amazonia and the Atlantic Forest, and 2) use forest habitat. For these species, we quantified the genetic data available in GenBank [33]. We also identified potential past connective routes between Amazonia and the Atlantic Forest by investigating the distribution maps for each species.

### Geographical data

Geographical distribution maps of forest mammalian species from Amazonia and the Atlantic Forest were compiled from the IUCN—International Union for Conservation of Nature [34]. We used the IUCN distribution data since these maps are created and verified by specialists based on occurrence records already checked and thus restrict species occurrences to areas with presumably suitable habitat where the species is known, following a precautionary principle to guide conservation efforts [35, 36]. Although these maps were designed for conservation purposes and recent studies suggest new methods for improving the IUCN maps and classifications [37, 38], these maps have proved to be an important source of information for many macroecological studies [39–44] and represent the most complete currently available species distribution maps for different mammal taxon.

To identify the mammalian species that occur in the two regions, Amazonia and the Atlantic Forest, the IUCN maps were overlaid on the Ecoregion maps [45] using the Amazonian and the Atlantic Forest limits, through the *gIntersection* function of the R package ‘rgeos’ v. 0.5.5 [46] in R v. 3.6.3 [47]. Subsequently, only species with occurrences in both Amazonia and the Atlantic Forest were selected. The predefined identifications based on the overlaid IUCN occurrence maps were revised using the annotated list of mammals in Brazil [48] since this reference agrees with current geographical and genetic data available in the databases used in this study.

### Habitat classification

We selected solely species that are associated with forests by accessing the IUCN information on species habitat use through the *rl_habitats* function of the R package ‘rredlist’ v. 0.6.0 [49]. We generated a scale of habitat preference for each species from forest specialist to generalist, as this is key information for studies about the connective routes between Amazonia and the Atlantic Forest. We based this scale of habitat preference on the detailed text about species’ habitat and ecology, available in the IUCN database [34] and additional literature reviews [50–56]. The criteria used for classifying the species according to habitat were as follows: 1) Strict forest specialists (SF), encompassing species that only occur in forests; 2) Species that prefer forest habitat (PF), encompassing species that use not only forested habitats but prefer these environments; or 3) Generalists (G) encompassing species that use both forests and open environments. Then, we used a Pearson’s Chi-squared test to assess the relationship between the species’ habitat preferences and the routes that they used through the *chisq*.*test* function in the R package ‘stats’ v. 3.6.3 [47].

### Genetic data

We compiled genetic data for each species from the Genbank database [33]. Data compilation was done in January 2020, by registering the amount of molecular data available (nucleotide sequences) for each species. The genetic data was used to assess the taxonomic representativeness (i.e., which taxonomic groups represented the highest availability of published genetic data) and, consequently, their potential usefulness in evaluating the past existence and use of connections between Amazonia and the Atlantic Forest.

Using the quantile function of the R package ’stats’ [47] we created categories to denote the availability of genetic data and assess the potential usefulness of the mammalian species. We considered species with one to 22 nucleotide sequences in Genbank to have low availability of genetic data; species with 23 to 74 sequences were considered to have regular genetic data; species with 75 to 225 sequences in Genbank were considered to have intermediate data availability; and species with 226 to over 1000 sequences were considered to have high data availability. Species without genetic data were not included in these categories, but are listed in the supplementary material (S1 Table). To compare the availability of genetic data among the different taxonomic groups, we also calculated the average number of sequences per species within each taxonomic order.

### Identification of potential connective routes

To identify the connective routes that mammals potentially have used between Amazonian and Atlantic forests, we first delimited the geographical areas of the connections using Olson’s ecoregion polygons overlaid on the ecoregion maps [45]. The area of the Eastern route was initially delimited using the boundaries of the Caatinga ecoregion, the transition areas between the Caatinga, the northern Cerrado and eastern Amazonia, the Babaçu Forest, and adjacent dry forests, which represent transition areas between Amazonia and the Atlantic Forest. The area of the Central route was selected using the limits of the Cerrado ecoregion (excluding the northern part, which was selected for the Eastern route). The area of the Western route was delimited using the boundaries of the Pantanal and the Chaco ecoregions, the transition areas between Amazonia and the Pantanal (such as Chiquitano Dry Forests), the southern Cerrado and the southwestern Atlantic Forest. For the final delimitation of these routes, we included additional potential areas based on the biogeographical [5–10], palynological [11–13], and geological [14] evidence of these past connections.

To explore how many species might have used each connective route, the delimited area for each route (Eastern, Central, and Western) was intersected with the species distribution maps using the function *gIntersection* in the R package ‘rgeos’ v. 0.5–5 [46]. Thereby, we quantified the total number of species associated with each route and the number of routes associated with each species. To visualize this result in the geographic space, we calculated the sum of rasters using the R package ‘raster’ v. 3.3 [57].

## Results

We compiled geographic distribution maps, information about habitat preferences, and genetic data for 127 mammal species occurring in both Amazonia and Atlantic Forest. The mammals belonged to nine taxonomic orders: Didelphimorphia (7), Pilosa (4), Cingulata (5), Perissodactyla (1), Cetartiodactyla (3), Primates (2), Carnivora (12), Chiroptera (84) and Rodentia (9). For the information per species see S1 Table. According to the IUCN, the geographic distribution of 113 of the species appear to be continuous between Amazonia and the Atlantic Forest, whereas the remaining 14 species present disjunct distributions (S1 Table).

**Fig 1:**
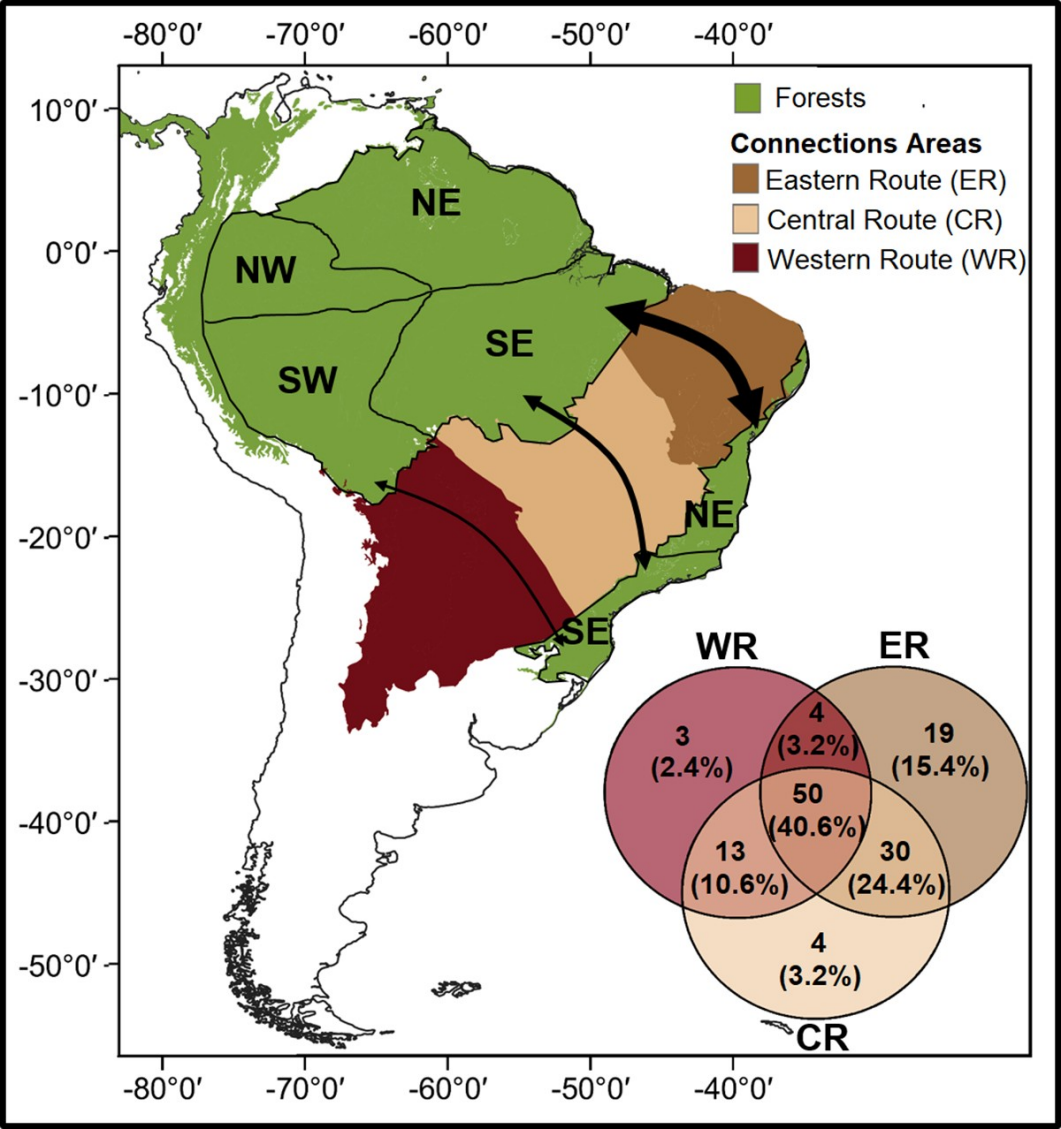
**Distribution of tropical moist forests in South America (in green) and potential past dispersal routes (in browns) between Amazonia and the Atlantic Forest.** Internal arrows represent connective routes between these forests through the Eastern route (dark brown), the Central route (light brown) and the Western route (reddish-brown). The width of the arrows represents the potential frequency by which the routes have been used in the past and is based on mammal species distributions. The coloured regions in the map were delimited using the Ecoregions shapefile from Dinerstein et al. (2017) licensed under CC-BY 4.0 (https://creativecommons.org/licenses/by/4.0/). The map was created in QGIS v.3.6.2 (https://www.qgis.org/) licensed under CC-BY-SA 3.0 (https://creativecommons.org/licenses/by-sa/3.0/https://creativecommons.org/licenses/by/4.0/). The Venn diagram shows the number of mammalian species that may have used the past connective routes between Amazonia and the Atlantic Forest, based on the IUCN geographical distribution maps.

**Fig 2:**
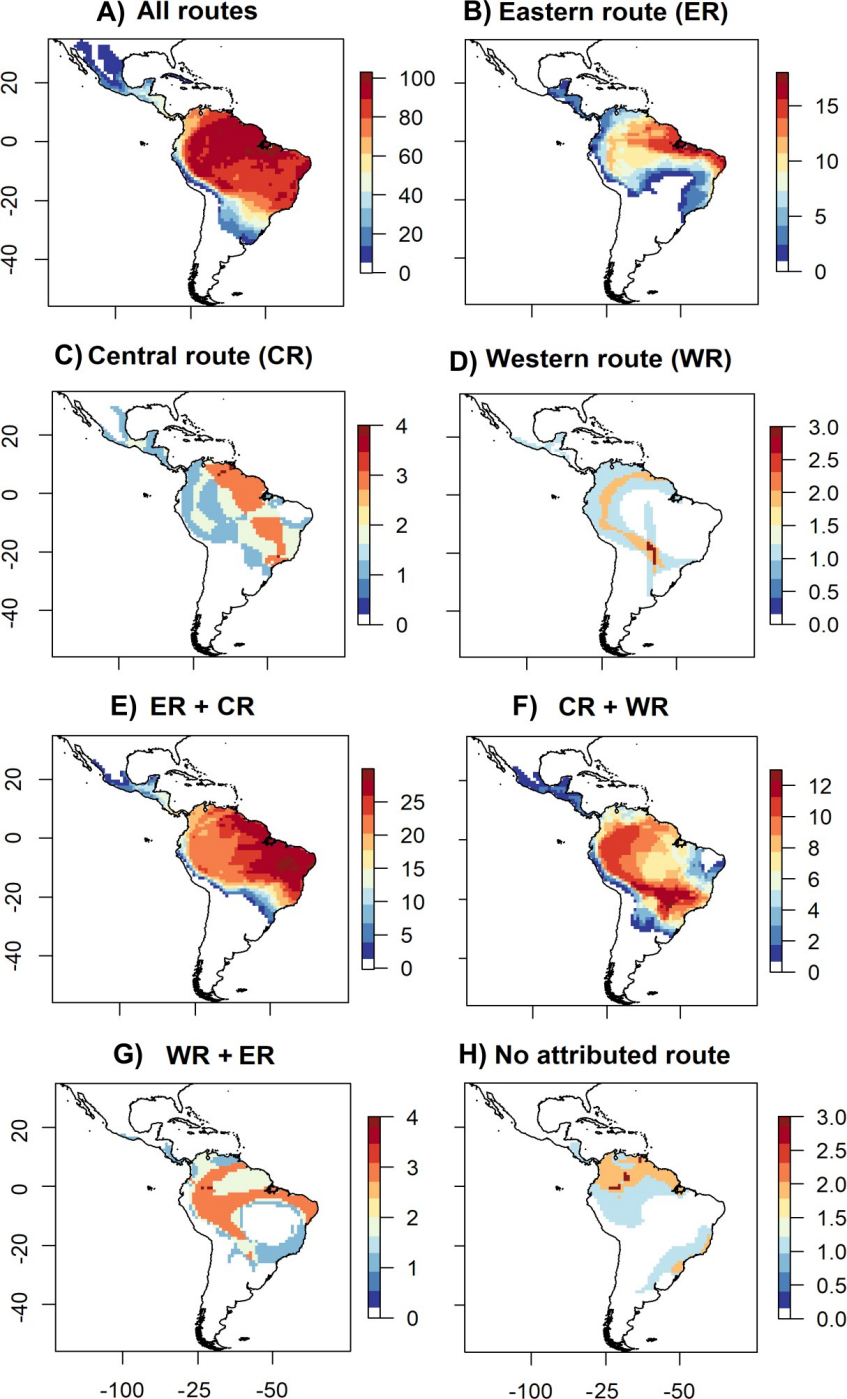
Overlap in mammal species distributions and tropical moist forest connective routes. The colour scale represents the number of species per pixel in the map. A) Overlap in species distributions with tropical moist forest habitat for all mammal species sampled in this study; B) Overlap in species distributions with the Eastern route; C) Overlap in species distributions with the Central route; D) Overlap in species distributions with the Western route; E) Overlap in species distributions with both the Eastern and Central routes; F) Overlap in species distributions with both the Central and Western routes; G) Overlap in species distributions with both the Western and Eastern routes; and H) Overlap in species distributions with no attributed route. Most species were associated with the Eastern route, followed by the Central route and finally the smallest number of species were associated with the Western route.

For four species it was not possible to attribute a potential connective route, as these present extremely disjunct distributions between Amazonia and Atlantic Forest (S1 Table). From the 123 species with attributed routes, 19 species were associated with the Eastern route only, four species were associated with the Central route only and three species were associated with the Western route only (Fig 1). The remaining 97 species were associated with more than one connective route. Of these, 50 species were associated with all three connective routes, 30 species were associated with both the Eastern and the Central routes, 13 species were associated with the Central and Western routes, and four species were associated with both the Eastern and Western routes (Fig 1). The distribution patterns of the species associated with each route, more than one route, or no attributed routes, can be seen in Fig 2.

**Fig 3:**
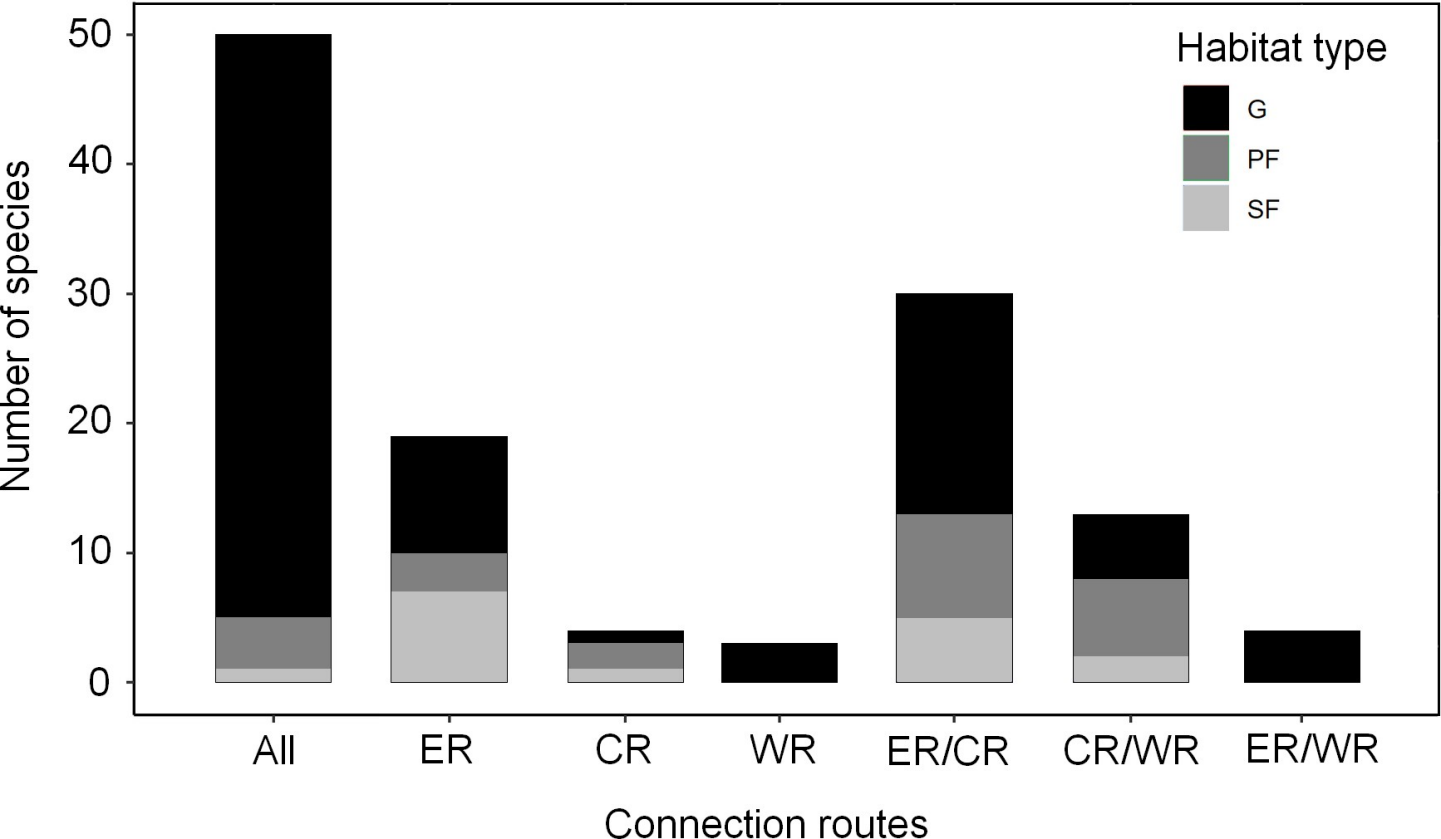
Number of mammalian species assumed to have dispersed by each of the connective routes between Amazonia and the Atlantic Forest. The routes are presented along the x-axis: Eastern route (ER), Central route (CR), and Western route (WR), and combinations of routes (ER + CR, ER + WR, CR + WR, and “All” for ER + CR + WR). The grayscale represents species habitat preferences where SF = Strict forest specialists, PF = Species that prefer forest habitat, and G = Generalists.

In terms of habitat use, 17 species were classified as strict forest specialists (SF), 24 species preferred forest habitat (PF) and 86 species were generalists (G). From the 19 species associated with the Eastern route, seven species were SF, three PF and nine were G (Fig 3). From the four species associated with the Central route, one species was SF, two PF and one was G, whereas the three species associated with the Western route were G (Fig 3). From the 30 species associated with both the Eastern and the Central routes, five were SF, eight PF, and 17 were G (Fig 3). From the 13 species associated with the Central and West routes, two were SF, six PF and five were G, whereas the four species associated with the Eastern and Western routes were G. Finally, from the 50 species associated with all three connective routes; one was SF, four PF and 45 were G (Fig 3 and S1 Table). The result of the chi-square test for the habitat preference was significant (Chi-squared2 = 35.255, df = 12, p-value = 0.0004). Thus, we reject the null hypothesis, which stated that habitat preference was independent from choice of connective route.

**Fig 4:**
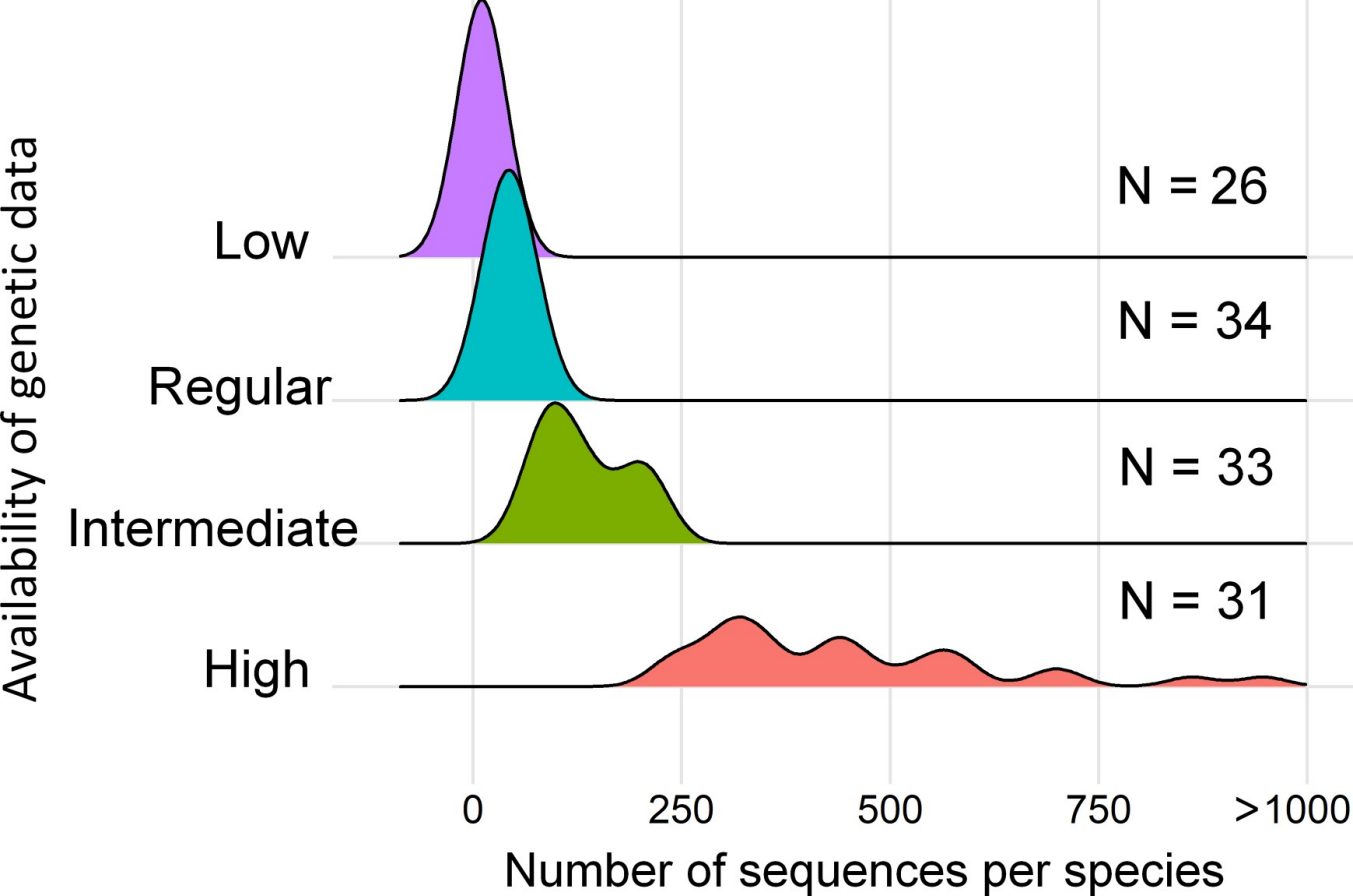
Availability of genetic data for mammalian species that occur in both Amazonia and the Atlantic Forest. Species with “Low” availability of genetic data have one to 22 sequences; Species with “Regular” availability of genetic data have 23 to 74 sequences; Species with “Intermediate” availability of genetic data have 75 to 225 sequences; and Species with “High” availability of genetic data have 226 to over 1000 sequences.

Most of the species identified in this study as potentially useful for assessing the connections between Amazonia and the Atlantic Forest have large amounts of genetic data available in the investigated database (high or intermediate availability of genetic data) including different molecular markers (Fig 4 and S1 Table for total number of available molecular data and Genbank access link for each species). Twenty-six of the investigated species showed low availability of genetic data in Genbank, 34 species show regular genetic data availability, 33 intermediate and 31 high availability of genetic data (Fig 4 and S1 Table).

**Fig 5:**
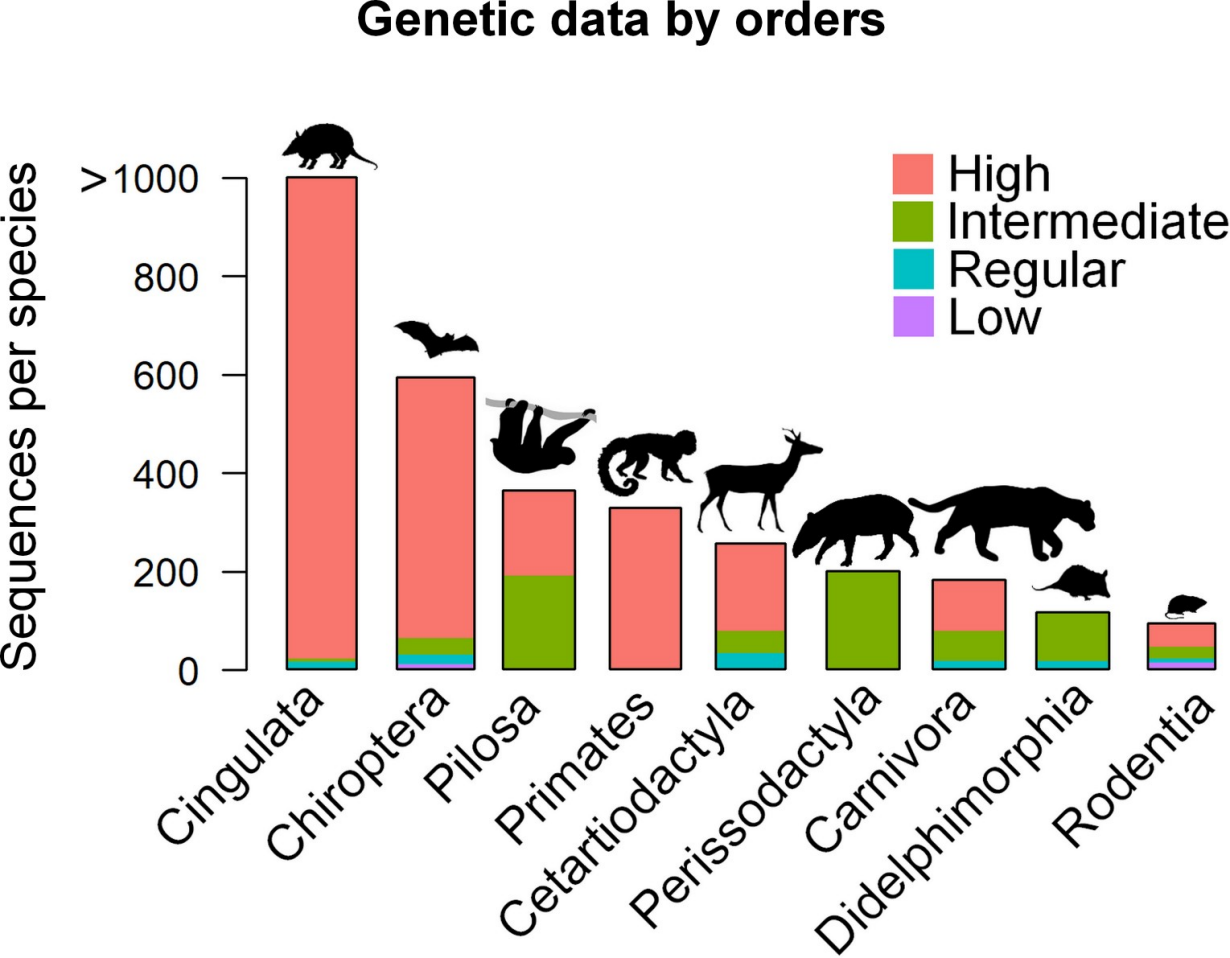
Availability of genetic data by order for species of mammals that occur in both Amazonia and the Atlantic Forest. The x-axis shows the mammalian orders and the y-axis shows the average number of sequences per species. Colours represent availability of genetic data. Animal silhouettes are reprinted from PHYLOPIC (http://www.phylopic.org/) under a CC-BY 4.0 license (https://creativecommons.org/licenses/by/4.0/), with permission from Michael Keesey, 2019.

Considering the orders, the Cingulata and Chiroptera contained most of the available genetic data (averaging 1,000 and 590 sequences per species, respectively; Fig 5), followed by the Pilosa, Primates, Cetartiodactyla, Perissodactyla (averaging 330, 300, 220, 200 and 190 sequences per species respectively; Fig 5). The Didelphimorphia and Rodentia also showed a considerable number of available nucleotide sequences (averaging 115 and 93 sequences per species, respectively: Fig 5). The availability of genetic data was high and intermediate for most orders. Some orders had regular data and few orders had low availability of genetic data (Fig 5 and S1 Table).

## Discussion

Here we present an unprecedented list of mammalian species with potential for investigating the past connections between Amazonia and the Atlantic Forest. Previous studies, including data compilations, analysed a limited number of mammalian species [10]. We were able to include 127 species of mammals currently occurring in both Amazonia and the Atlantic Forest to add information about the potential past connections between these two ecoregions. We compiled information about the species’ distributions, habitat preferences, and the quantity of genetic data available. We show that mammal habitat use is significantly associated with their respective dispersal routes and that three routes have probably connected the two rainforest regions in the past. The species list compiled herein has the potential to subsidize future mammal phylogenetic and phylogeographic studies and to shed light on the temporal and spatial use of the connections between Amazonia and the Atlantic Forest in relation to the ecology and evolution of South American mammals.

By overlaying the present-day geographic distribution maps of the candidate species over the three proposed historic routes between Amazonia and the Atlantic Forest we show that most of the 127 mammalian species were associated with the Eastern route. These species also evidenced strikingly continuous distributions across the dry diagonal between the forest habitats–albeit biased toward the coast. Thus, their present-day geography provides a compelling indication that the Eastern route, with its possible access to coastal habitats, may have been preferred over the other routes. The Central route was associated with the second most species, and, contrary to previous suppositions, the Western route was associated with the fewest species. Because most data previously used to test the existence of the connective routes over time came from interspecific molecular evidence from a few species, and because most species split before the Plio-Pleistocene (~5 mya to 12 thousand years ago) [58], such data may have biased results toward older routes, e.g., toward the Western route which would have occurred for the first time at least between ~30 to 20 mya in the Oligo-Miocene [5, 8, 10]. In contrast, studies on paleo vegetation, pollen data and biogeographic approaches indicate that several past connections through the Eastern route could have occurred during the Pleistocene (~2.5 mya) [11–14, 21, 29, 59, 60]. Hence, these insights highlight a need of more intraspecific studies that look at the genetic divergence within multiple taxa since such studies may shed light on the complexity of the evolution and existence of the connections between Amazonia and the Atlantic Forest over time [61–64].

The Eastern and Western routes were associated with species that had a great variety of habitat preferences. Thus, considering that the current distribution patterns of most species reflect past dispersions, their past use of the connective routes seems related to the environmental heterogeneity of these areas. For instance, the present-day geographic distribution of most generalist species aligned with the Eastern route which passed through the Caatinga and the Cerrado/Caatinga transition, a region of high environmental heterogeneity [65, 66]. In contrast, the Central route was predominantly associated with strict forest specialists or species that prefer forest habitat. As highlighted by Costa [6], the forest environments in the Cerrado ecoregion represent relic forest fragments from historic ecological corridors [67], which allow forest species to be present in the region. Hence, our results highlight the probable importance of the Central route as an independent past connection for several forest specialist species.

Although the Central route had already been considered a potentially independent route in some studies [5, 6, 23, 25], other studies based on regional climatic similarities, considered only two major connections between Amazonia and the Atlantic Forest [8, 10, 18]. Reconciling these views, one may argue that we found two major dispersal patterns with more species distributions through the eastern connections (i.e. through the northern Central route and the Eastern route combined) than through the western connections (i.e. through the southern Central route and the Western route combined). Still, our findings point to three independent connection routes. Hence, we believe that looking past present-day climatic similarities to include past environmental changes [11–13] and analyses of species with a wider range of habitat preferences could bring new insights about the past prevalence and use of the connections between Amazonia and the Atlantic Forest.

Many of the species that show extreme disjunct distributions, such as the four bat species in this study, may represent either sampling deficiencies or even cryptic diversity [68]. Hence, sampling deficiencies in the north-eastern dry diagonal, in addition to the non-inclusion of specimens deposited in museum collections or published records of species in this region [24] may have led to faulty estimates of the number of potential taxa that could have spread along the Eastern route. As new records appear with increased sampling effort through systematic biogeographic studies in this region, future reassessments will thus, almost certainly, find additional species, which evidence past dispersions along the Eastern route [69–73, 76]. For example, several recent studies have presented new records of common forest species, such as for marsupials [74–77] and rodents [78, 79] along the Eastern route. Hence, updating the known distributions of these species, and others, are crucial for correctly assessing the past connective routes between Amazonia and the Atlantic Forest.

According to the IUCN maps, used in our study, many mammalian species show continuous present-day distributions between Amazonia and the Atlantic Forest through deciduous and semi-deciduous forests in the interior of the dry diagonal. However, phylogeographic studies have revealed that some of these species, with seemingly continuous distributions, actually consist of currently isolated populations [e.g., 80]. Moreover, many isolated populations show evolutionary well-structured lineages with significant genetic divergence, suggesting that the taxonomic status of these species needs revision as they could represent species complexes [e.g., 6, 52, 62, 80]. Hence, further phylogeographic studies are necessary if we are to reveal whether these mammalian populations are indeed connected or isolated in function of the current fragmentation patterns of the South American forests. Furthermore, such studies could help identify population isolations of anthropic origin, for example in the highly fragmented Atlantic Forest remnants and the Amazonian arc of deforestation.

Indeed, as we continue to lose forest habitat, the landscape becomes increasingly fragmented. Unfortunately, the environmental protection system has hitherto failed to connect the forest environments between Amazonia and the Atlantic Forest [81]. Due to the stance and actions of the current Brazilian, federal government, which has inspired serious negligence of Brazilian environmental laws [82], actions to increase the connectivity between the two ecoregions are currently unlikely. Given the relationship that we observe between species’ habitat preferences and their associations with past connection routes this is unfortunate since continued forest loss and habitat fragmentation could have dire consequences for the populations along these dispersal pathways.

The availability of genetic data for our 127 species, revealed that many of them would serve for assessing the existence and importance of the past connections between Amazonia and the Atlantic Forest. Depending on the geographic distribution of data, species with regular to high availability of genetic data could serve this purpose. Hence, this initial compilation can be extremely useful in facilitating more detailed evaluations and future phylogeographic explorations of the past connections between Amazonia and the Atlantic Forest. For instance, *Marmosa demerarae* and *Marmosa murina* had regular (n = 47 sequences) and intermediate (n = 77 sequences) genetic availability, but adequate geographic distribution of samples (totalling 31 and 39 localities along their respective ranges) and hence serve for investigating the Eastern and Central routes respectively (Machado et al., in prep.). An example of a species with low genetic availability (n = 13 sequences) but good geographic coverage (11 localities) was *Caluromys philander*. Increasing the number of sequences per locality would thus render *C*. *philander* a good candidate for investigating the Central route. For other species increasing the geographical coverage rather than the number of sequences per locality might be more important. Although most mammalian species had high availability of genetic data, it is noteworthy that the two groups with the highest availability of genetic data are groups of high public health interest [83]. This result therefore showcases the extreme importance of making scientific data available for the development of new studies that go beyond the focus areas of the projects that generated the data.

In conclusion, we present information about the distributions, habitat preferences, and availability of genetic data for 127 mammal species currently occurring in both Amazonia and the Atlantic Forest. These data will certainly help future phylogeographic and phylogenetic studies to unravel the evolutionary history of these mammals and the past connections between Amazonia and the Atlantic Forest. Furthermore, the initial data exploration presented herein shows that more species presently occur along the Eastern route. Thus, unlike previously thought, we hypothesise that the Eastern route may have been used by more taxa and may have occurred more frequently than the other routes in the recent past. However, further data curation is needed to test this hypothesis and it remains to be seen to what extent the present-day geography of the 127 mammals can inform us about the past dynamics of these megadiverse, and highly threated, forests.

## Supporting information

S1 Table Potential mammal species for investigating the past connections between Amazonia and the Atlantic Forest with geographic, taxonomic, ecological and molecular data (including GenBank accession data). (XLSX)
