# Supplementary material for: Potential mammalian species for investigating the past connections between Amazonia and the Atlantic Forest
Source: PLoS One. 2021 Apr 9;16(4):e0250016. doi: 10.1371/journal.pone.0250016 (PMC8034742; doi:10.1371/journal.pone.0250016)
Supplement: Attachment — Submitted filename: Response_to_Reviewers.docx [file pone.0250016.s002.docx]

Dear Paulo Corti

Editor in Chief

PLOS ONE

We were happy to hear that you found our manuscript entitled: “**Potential mammalian species for investigating the past connections between Amazonia and the Atlantic Forest**” (PONE-D-20-40510) to be of interest. We found the comments and suggestions put forward during the review process to be most helpful and we thank you for the opportunity to submit this revised version of our work.

Please see below for our responses (in blue) to each point raised by the editor and the reviewers (in black). Line numbers refer to the "clean" version of our manuscript (i.e. without track-changes).

On behalf of all other co-authors,

Camila D. Ritter.

**Point-by-point**

Editor’s requests:

1. “Please ensure that your manuscript meets PLOS ONE's style requirements, including those for file naming.” Reply/Action taken: We have checked the formatting style of PLOS ONE and formatted our manuscript according. We also made some additional changes to better meet PLOS ONE's requirements. For example, we chose to remove “Table 1” from the manuscript and keep it as supplementary material (“S1Table”), since keeping the table in the body of the text would have resulted in six pages and thus, an immense cut in the text. But we are open to revert this decision, in case the editor should prefer to keep S1 Table as part of the main article.

2. “We note that you have stated that you will provide repository information for your data at acceptance. Should your manuscript be accepted for publication, we will hold it until you provide the relevant accession numbers or DOIs necessary to access your data. If you wish to make changes to your Data Availability statement, please describe these changes in your cover letter and we will update your Data Availability statement to reflect the information you provide.” Reply/Action taken: We are sorry for the confusion. We meant to say that we used already freely available data from the repository databases, GenBank, IUCN, and Dinerstein et al. (2017) shapefiles. Thank you for drawing our attention to this mistake.

3. “We note that Figures 1 and 6 in your submission contain map images which may be copyrighted. All PLOS content is published under the Creative Commons Attribution License (CC BY 4.0), which means that the manuscript, images, and Supporting Information files will be freely available online, and any third party is permitted to access, download, copy, distribute, and use these materials in any way, even commercially, with proper attribution. For these reasons, we cannot publish previously copyrighted maps or satellite images created using proprietary data, such as Google software (Google Maps, Street View, and Earth).” Reply/Action taken: We used shapefiles that are under CC BY 4.0 license. In Figure 4 (now Fig 5) we used animal silhouettes images from PHYLOPIC provided under CC BY 4.0 license. All citations and links are now provided in the figure legends.

While revising your submission, please upload your figure files to the Preflight Analysis and Conversion Engine (PACE) digital diagnostic tool, https://pacev2.apexcovantage.com/. PACE helps ensure that figures meet PLOS requirements. To use PACE, you must first register as a user. Registration is free. Then, login and navigate to the UPLOAD tab, where you will find detailed instructions on how to use the tool. If you encounter any issues or have any questions when using PACE, please email PLOS at figures@plos.org. Please note that Supporting Information files do not need this step. Reply/Action taken: We have uploaded our figures using PACE and checked that they meet the PLOS requirements.

Reviewer #1:

1. “Since the glacial forest refugia hypothesis of Haffer (Science 1969) a pleothora of studies have addressed the history of the extension of Amazonian rainforest. The history of Atlantic rain forest was often addressed in tandem. Due to the few available deep-time pollen records the dynamic history of Amazonian rainforest is still largely unknown (a ‘reasonable guess’ in the papers by M. Bush et al.) and still subject to much speculation. The history of the Atlantic rain forest is better known. In fact, Hoorn et al. (Science 2010) showed that the immense biodiversity originates from much older age as the Pleistocene, and that ice age cycles do not play an important role to explain high levels of biodiversity in Amazonia. This makes it interesting how old connecting routes should be. Are authors hinting to connecting routes of Pleistocene age? Perhaps even the most recent part of the Pleistocene? I am surprised that the Introduction does not place this study in a Pleistocene context providing arguments why a search for corridors could be potentially a contribution to this debate at large.” Reply/Action taken: We agree regarding the importance of anchoring this study in time. We have therefore reviewed the introduction to include more information on the origins of the tropical rainforests in South America and their changes over time, which are important for understanding the connections between Amazonia and the Atlantic forest. The introduction now reads: “The origin of the tropical rainforests in South America is dated to at least 65 million years ago (mya) [15-17]. Since then, these forests have undergone several changes, expanding and retracting. From the Oligocene (~ 23 mya) to the Pliocene (~ 3 mya), successive tectonic events led to the Andean uplift, restricting the entry of rainfall from the Pacific into the interior of the continent resulting in a drier climate with a forest reduction and the expansion of savannas, giving rise to the dry diagonal, and, consequently, the separation of Amazonia and the Atlantic Forest [15, 18-20]. Furthermore, there is evidence of more recent forest expansions and retractions caused by Quaternary climatic fluctuations, such as glacial cycles during the Pleistocene and recurrent periods of extreme rainfall during the Holocene [11-14]. Indeed, the high rates of vegetation cover changes in the dry diagonal and the Atlantic Forest [21, 22] suggest not just ancient but also recent connections and disruptions between Amazonia and the Atlantic Forest [9]” (lines 50-61).

2. “This study follows an interesting way of data analysis to recognize connecting routes between both forested areas. Basically I am surprised that the authors talk about ‘past connective routes’ (line 234) and do not a single effort to state more precisely ‘past’ along the time scale. However, with the history of Amazonian rain forest so poorly understood it is perhaps difficult to do a better job?” Reply/Action taken: The reviewer correctly observes that it is a difficult job to anchor the connective routes between Amazonia and the Atlantic forest in time, but by creating a list over mammalian species that contains geographical, ecological, and genetic information we present a first and important step in this direction. To elucidate the history of the past routes and precisely place them along the timescale would however require phylogenetic and phylogeographic analyses that are beyond the scope of this paper. Yet, we agree that this will be key for understanding the evolution and dynamics of Amazonia and the Atlantic Forest. Therefore, in the introduction, as shown above, we have made an effort to better elaborate on what is known about the time scale of these connections, as well as possible sample biases.

In addition, we added the following to the discussion: “Because most data previously used to test the existence of the connective routes over time came from interspecific molecular evidence from a few species, and because most species split before the Plio-Pleistocene (~5 mya) [60], such data may have biased results toward older routes, e.g., toward the Western route which would have occurred for the first time at least between ~30 to 20 mya in the Oligo-Miocene [5, 8, 10]. In contrast, studies on paleo vegetation, pollen data and biogeographic approaches indicate that several past connections through the Eastern route could have occurred during the Pleistocene (~2.5 mya) [11-14, 21, 29, 58-59]. Hence, these insights highlight a need of more intraspecific studies that look at the genetic divergence within multiple taxa since such studies may shed light on the complexity of the evolution and existence of the connections between Amazonia and the Atlantic Forest over time [76-79].” (lines 297-307).

In the discussion we also state clearly that our objective with this study and the species list is to: “subsidize future mammal phylogenetic and phylogeographic studies to shed light on the temporal and spatial use of the connections between Amazonia and the Atlantic Forest in relation to the ecology and evolution of South American mammals” (lines 290-292).

3. “Although I am very much focused on botany, I follow the methods, but I cannot assess the methods used critically. Using Olson’s ecoregion polygons (line 152) is a reasonable approach.” Reply/Action taken: We used Olson’s ecoregion polygons to delimit the extent of the routes. This ecoregion database is the most complete database for ecoregions of the world [45]. Furthermore, our route regions agree with the previous literature [5, 6, 23, 25].

4. “As mentioned in the Discussion section (lines 210-211) this study reflects a new compilation of ever expanding data and results shows an incremental step forwards. Indeed, results may serve as a basis for future biogeographic studies considering different mammalian taxonomic groups (lines 98-99). As such this study is well designed, well presented and timely. I am not sure if the incremental step forwards is groundbreaking. Is this study able to bring more focus in the wide array of discussions about the history of Amazonian and Atlantic forest areas? The authors have not addressed this issue, making this fine study to a mammalian-specific palaeo-biogeographical contribution.” Reply/Action taken: Building on our data compilation future studies will be able to bring new insights that contribute to the wide array of discussions about the history of Amazonian and Atlantic forest areas, for example through phylogenetic and phylogeographic analysis that are beyond the scope of this study. To compile mammalian geographical, ecological, and genetic data is difficult and time consuming, therefore we decided to make this list available to assist researchers interested in South American mammal biogeography. We appreciate this reviewer's comment, and we restructured the discussion to be clearer and more direct about our findings.

5. “References: are not consistent (373. 377, 398)” Reply/Action taken: We have checked all references to ensure consistency in this new version.

6. “Figure 1: make a better difference between the three colors of brown.” Reply/Action taken: We thank the reviewer for the suggestion. We now use higher contrast colours and we have checked all figures for colour-blind mode.

7. “Figure 2: grey scale is unclear.” Reply/Action taken: We now use a more contrasting grey scale, and this figure is now the Fig 3 to better structure the order of mention of the results.

8. “Figures 1 and 5 could easily be combined.” Reply/Action taken: We thank the reviewer for the suggestion. We merged figures 1 and 5.

9. “In conclusion this is a fine study using an updated dataset and reaching relevant conclusions in a longstanding debate. This study is quite mammalian specific. I am wondering if the authors have tried to let results integrate in the palaeo-biogeographical discussion of Pleistocene Amazonian rainforest dynamics.” Reply/Action taken: We now contextualize the time scale of the proposed routes in both the introduction and the discussion by integrating information from palaeo-biogeographical studies. However, we do not directly discuss the Pleistocene Amazonian rainforest dynamics in depth because it is not the objective of the study and because the Pleistocene cycles appear to have been less pronounced inside Amazonia [20]. Indeed, the environmental stability is proposed to have much been greater in Amazonia throughout the late Quaternary, whereas there were high rates of changes in vegetation cover in the dry diagonal and the Atlantic Forest [21, 22]. We do however elaborate on the impacts of the Pleistocene dynamics on forest expansions and retractions in the dry diagonal leading to the establishment of connection routes between Amazonia and the Atlantic Forest.

Reviewer #2:

Overall recommendation:

1. “While this is a worthy exercise that may yield new insights into the paleo-ecological question, the paper as written does not present sufficient evidence to support the primary conclusion – that the NW pathway is most important – which runs contrary to the bulk of previous literature on the topic. The authors have assembled a nice baseline of information to support an intriguing new hypothesis, but major revisions to the framing of the paper would be required to cast this current work as a scientific finding.” Reply/Action taken: We agree and therefore reviewed the text to strengthen our findings with arguments in accordance with the literature. We highlight that the limited number of species used in previous studies could have biased their conclusions but also stress that further studies are necessary to test this proposition and our hypothesis about the importance of the route which we refer to as the Eastern route. We thus hope to clarify that also we call for further studies, which are necessary if we are to shed light on the past connections between Amazonia and the Atlantic Forest. It is for this reason that we make our mammal list with ecological, geographical and genetic data widely available.

Additional comments:

2. “There is a substantial body of literature on this particular question – the authors would do well to more fully summarize the question at hand as well as existing scholarship so that readers not as familiar can better engage with their analysis. Although plenty of references are cited, a bit more set up is warranted. The use of unmodified range polygons from IUCN is problematic for a precise vector-based analysis due to known uncertainties and omission/commission errors (see Brooks et al. 2019 <https://doi.org/10.1016/j.tree.2019.06.009>; Jung et al. 2020 <https://doi.org/10.1038/s41597-020-00599-8>).” Reply/Action taken: We understand the concern in relation to the IUCN expert maps, since there is great disbelief in this data for other taxonomic groups. However, these maps are created and verified by specialists based on occurrence records, with the predicted presence being restricted to areas with adequate habitat where the species is known and adhering to the precautionary principle that guides conservation efforts. Although these maps were designed for conservation purposes, they are widely used. In addition, they have been extensively updated to consider taxonomic revisions and fragmentation of mammal distributions. Using occurrence maps generated by species-level experts is a better option for larger-scale studies than using occurrence records, due to the extensive errors associated with the latter, such as problems with false positives due to erroneous taxonomic identifications and errors in coordinates. Finally, studies testing the use of occurrence records versus IUCN maps have shown that specialized knowledge provides more robust predictions and/or correlated results. Thus, we justify our use of the IUCN data in the following way: “We used the IUCN distribution data since these maps are created and verified by specialists based on occurrence records already checked and thus restrict species occurrences to areas with presumably suitable habitat where the species is known, following a precautionary principle to guide conservation efforts [35, 36]. Although these maps were designed for conservation purposes and recent studies suggest new methods for improving the IUCN maps and classifications [37, 38], these maps have proved to be an important source of information for many macroecological studies [39-44] and represent the most complete currently available species distribution maps for different mammal taxon”. (lines 118-125).

3. “Likewise, attribution of a particular dispersal route base on range intersection with precise ecoregion boundaries to me is not sufficient to assign a particular route at the exclusion of the other two – particularly when identifying dispersal routes through varying climate, land cover, ecoregion boundaries and species ranges back to 120,000 ybp. The authors, at a minimum, would need to examine the sensitivity of different ecoregion boundaries to delineate potential routes, discuss the paleo-ecology that may have variably facilitated the different routes and offer potential explanations/reasons that this evidence is sufficient to reconsider the literature as to the relative importance of routes.” Reply/Action taken: We consider the regions previously described in the literature. These studies cover not only molecular data, but also geological, climatic and palaeoecological data. The ecoregions' shapefile was used as an initial base for the delimitation of the routes, but the final result of the delimited area being a mixture of information from areas previously described in the literature based on molecular data, geological, climatic and palaeoecological data complemented ecoregion data. Now, figure 1 includes the areas delimited by us for each route. Although we consider these areas an adequate starting point for investigating the past connections between Amazonia and the Atlantic forest, further studies are needed to confirm our results. Throughout the discussion, we therefore highlight that this initial compilation is to serve as basis for future studies.

4. “The list of mammal species was queried against GenBank records, but there was no subsequent prioritization of species based on the quantity of sequences – would all be used? Just those with high data availability? The authors note: “However, it is important to highlight that, although many of these species have a large amount of available genetic data, it is common for many of the nucleotide sequences to contain missing information, which could render them unfit for use.” – it seems this next step would be necessary to include the GenBank availability in a species screening exercise.” Reply/Action taken: Yes, further analysis and curation of the genetic mammal data are necessary to investigate the past connections between Amazonia and the Atlantic forest. Next steps would include GenBank sequences curation. This would require quite some effort for the 127 species (more than 150,000 sequences in total) since, unfortunately, Genbank does not provide any location-based data filtering tools (which would be important for future phylogeographic studies). In this study our goal was simply to provide a list of mammalian species with a potential for investigating the historic South Americas forest dynamics and their ecologic, geographic and genetic data. From our own experience, this list was essential to select species to be used in our research. Therefore, we want to share these data to facilitate for future phylogeographic studies. However, the inclusion of species in such future studies, would depend on their exact research questions.

5. “The maps in figure 6 are difficult to reconcile vs. the known richness patterns of the Atlantic forest. For example, it appears as though in panel B – the majority of species ascribed to NE route only intersect the very northern portion of the Atlantic forest and there is not much richness of those species throughout the remainder of the Atlantic forest ecosystem. This pattern at least should be noted in the results/discussion. In panel D – only 1 species looks to have any range in the Atlantic forest – doesn’t that violate the first screening criterion?.” Reply/Action taken: We appreciate the concern expressed by the reviewer and acknowledge that the previous figure may not have made our data justice. We therefore reworked the figure to include all species distribution patterns (i.e., also species that evidenced use of multiple routes). Including all species led us to identify an error in the tabulation of the data (i.e., an erroneous combination of routes), which we have now corrected for. However, these changes have not changed the main results (i.e., the pattern found has remained the same). This correction resulted in an increase in the number of species that evidence past use of the Eastern route, and with that, the results now show more species that occur throughout the entire Atlantic Forest, which can be seen in the new figure 2 (we changed the order of the figures to better structure the results). As for the species in panel D, all 3 species that occur on that route reach the limits of the Atlantic Forest. Due to little overlap in the distributions of these species, it is apparent that only one of them reaches into the interior of the Atlantic Forest. Two of them occur in this biome in larger areas (one occurring up to the South of the Atlantic Forest and another to the Central Atlantic Forest) and one of them occur at the edge of the Atlantic Forest (in the forests of the Paraná). Considering that there are forests that extend into the ecotone between the Atlantic Forest and the other biomes (for example, extensions of humid and seasonally humid forests in the north and northeast of Argentina) it would be prudent not to exclude this species from the list.

6. “There are minor grammatical and typographical errors throughout -- I am confident most/all would be caught in a revision.” Reply/Action taken: We apologize for these mistakes and carefully checked the text to avoid any errors in this new version.

7. “The authors clearly have deeper insights and ideas for lines of inquiry into this question than are presented in this manuscript (as evidenced by some interesting but untested ideas in the discussion). As mentioned in the overall recommendation, both the objectives of this manuscript and the application of this dataset to future studies needs to be more precisely defined. I wish the authors all the best in their continued investigation into this question!” Reply/Action taken: We thank the reviewer for this comment. Indeed, we created this species list to select potential mammal species for investigating the connections between Amazonia and the Atlantic Forest in other manuscripts. As collecting and filtering all data was hard and time consuming, we decided to share this list as an open access manuscript to help other researchers interested in using mammals as study objects in phylogeographic and phylogenetic studies to investigate past dispersal and forest dynamics.
