# Supplementary material for: Potential mammalian species for investigating the past connections between Amazonia and the Atlantic Forest
Source: PLoS One. 2021 Apr 9;16(4):e0250016. doi: 10.1371/journal.pone.0250016 (PMC8034742; doi:10.1371/journal.pone.0250016)
Supplement: Attachment — Submitted filename: Response_to_Reviewers_R2_final.docx [file pone.0250016.s003.docx]

Dear Dr. Paulo Corti

Academic Editor

PLOS ONE

Thank you for your careful consideration of our manuscript entitled: “**Potential mammalian species for investigating the past connections between Amazonia and the Atlantic Forest**” (PONE-D-20-40510). Here we provide a second revision of our manuscript and we hope that the current version will fully meet the PLOS ONE publication criteria.

Please see below for our responses (in blue) to each point raised by the editor and the reviewers (in black). Line numbers refer to the "clean" version of our manuscript (i.e., without track-changes).

On behalf of all co-authors,

Camila D. Ritter.

**Point-by-point**

Reviewer #1:

1. “I am fine with the improvements of the manuscript and the explanation.
   Note: I found the reference of Hoorn et al. (2010) with an incomplete list of authors (line 454).”

Author’s response:

Thank you for your attention. We corrected and standardized all references so that references with more than six authors read “et al.” from the sixth author on – thus, following the journal’s online publication guideline.

Reviewer #2:

2. “I thank the authors for their attention to my previous comments and appreciate their responses. However, I still feel that the primary conclusion of the manuscript – that the Eastern route is/was most commonly used – is not supported by the evidence presented. The authors do well to caveat their result in the discussion, but the conclusion remains plainly stated in the abstract (separately it appears the previous nomenclature for the dispersal routes remains in the abstract – i.e. ‘northeastern’ vs. ‘Eastern’). What the authors have found is that the present-day geography of the mammal species found in both the Amazon and the Atlantic Forests points to more species in the northeastern portion of the dry diagonal (and adjoining forested habitats). It remains to be seen if this present-day geography can offer any conclusions as to the paleo dispersal routes. The authors state in their response “Throughout the discussion, we therefore highlight that this initial compilation is to serve as basis for future studies.” I am recommending minor revisions to properly contextualize the conclusion of the most commonly used dispersal routes as a preliminary geography-based finding throughout – but especially in the abstract.”

Author’s response:

Thank you for these suggestions. We edited parts of the abstract (lines 29-30 and 37-41) to better clarify the study's findings and the limits of the interpretations. We also edited some excerpts throughout the text to strengthen our findings (lines 291, 300, 302-306, 320-323, 355, 378-379, 381-390, 396, and 400-405). Finally, we changed the names of the routes in the abstract (line 26) to standardize the nomenclature throughout the text.

3. “I find the authors’ response to the question of the Genbank records as a means to further prioritize this list to be unsatisfactory. If the intent of this list is to guide future investigation into this question of dispersal route, wouldn’t it make sense to hear from the authors as to what level of genetic information is sufficient or at least more preferable? Indeed, it depends on the research question, but surely the authors have recommendations for species among the 127 assessed as to which are more ripe for immediate future study and/or if there are species that could be strategically targeted for additional sampling to help confirm/deny a particular route.”

Author’s response:

We appreciate the reviewer comment and now specify that “*Depending on the geographic distribution of data, species with regular to high availability of genetic data could serve*” for investigating the past connection routes (lines 379-380). To exemplify this we give details about three species that could either be used immediately or after strategic sampling, to investigate the Eastern and Central routes, see lines 383-391: “*For instance, Marmosa demerarae and Marmosa murina had regular (n = 47 sequences) and intermediate (n = 77 sequences) genetic availability, but adequate geographic distribution of samples (totalling 31 and 39 localities along their respective ranges) and hence serve for investigating the Eastern and Central routes respectively (Machado et al., in prep.). An example of a species with low genetic availability (n = 13 sequences) but good geographic coverage (11 localities) was Caluromys philander. Increasing the number of sequences per locality would thus render C. philander a good candidate for investigating the Central route. For other species increasing the geographical coverage rather than the number of sequences per locality may be more important”.* Additionally, we have included both text and Fig 5 results for availability of genetic data by order, from lines 277-279: *"The availability of genetic data was mostly high and intermediate for most orders. Some orders had regular data and few orders had low availability of genetic data".* We also edited the count for categorized species with low availability of genetic data, so that these vary between one and 22 sequences (line 160), thus removing species without genetic data from this category. Species without genetic data are listed in the supplementary material with “absent” genetic availability (S1 Table), and consequently, we edited Fig. 4 to reflect this exclusion of species with “absent” genetic availability. You may also note that we changed the names of the categories to “low”, “regular”, “intermediate” and “high”, since we felt that these were more adequate.

4. “The maps in Fig 6 are much improved – and I thank the authors for their explanation (which in retrospect makes perfect sense). Thinking further, I am struck by how continuous the distributions of species attributed to the Eastern route are across the dry diagonal between the forest habitat – albeit biased toward the coast. This is actually a rather compelling argument that the Eastern route may have been preferred (possible access to coastal habitats en route)!

   My best wishes to the authors”

Author’s response:

We thank the reviewer for his/her comment. We added the following to the discussion (lines 303-307): “*These species* [attributed to the Eastern route] *also evidenced strikingly continuous distributions across the dry diagonal between the forest habitats – albeit biased toward the coast. Thus, their present-day geography provides a compelling indication that the Eastern route, with its possible access to coastal habitats, may have been preferred over the other routes*”.
