# Supplementary material for: Potential mammalian species for investigating the past connections between Amazonia and the Atlantic Forest
Source: PLoS One. 2021 Apr 9;16(4):e0250016. doi: 10.1371/journal.pone.0250016 (PMC8034742; doi:10.1371/journal.pone.0250016)
Supplement: Peer review history

# Peer review: Decision Letter 0

**DOI:** 10.1371/journal.pone.0250016.r001

**Authors:** Paulo Corti

5 Feb 2021

PONE-D-20-40510

Potential mammalian species for investigating the past connections between Amazonia and the Atlantic Forest

PLOS ONE

Dear Dr. ritter,

Thank you for submitting your manuscript to PLOS ONE. After careful consideration, we feel that it has merit but does not fully meet PLOS ONE’s publication criteria as it currently stands. Therefore, we invite you to submit a revised version of the manuscript that addresses the points raised during the review process.

Pay attention to comments of both reviewers, but specially reviewer #2. This reviewer has highlighted important issues regarding your conclusions to support raised hypothesis. 

Please submit your revised manuscript by Mar 22 2021 11:59PM. If you will need more time than this to complete your revisions, please reply to this message or contact the journal office at plosone@plos.org. When you're ready to submit your revision, log on to https://www.editorialmanager.com/pone/ and select the 'Submissions Needing Revision' folder to locate your manuscript file.

Please include the following items when submitting your revised manuscript:

- A rebuttal letter that responds to each point raised by the academic editor and reviewer(s). You should upload this letter as a separate file labeled 'Response to Reviewers'.
- A marked-up copy of your manuscript that highlights changes made to the original version. You should upload this as a separate file labeled 'Revised Manuscript with Track Changes'.
- An unmarked version of your revised paper without tracked changes. You should upload this as a separate file labeled 'Manuscript'.

If you would like to make changes to your financial disclosure, please include your updated statement in your cover letter. Guidelines for resubmitting your figure files are available below the reviewer comments at the end of this letter.

If applicable, we recommend that you deposit your laboratory protocols in protocols.io to enhance the reproducibility of your results. Protocols.io assigns your protocol its own identifier (DOI) so that it can be cited independently in the future. For instructions see: http://journals.plos.org/plosone/s/submission-guidelines#loc-laboratory-protocols

We look forward to receiving your revised manuscript.

Kind regards,

Paulo Corti, Ph.D.

Academic Editor

PLOS ONE

Journal requirements:

When submitting your revision, we need you to address these additional requirements.

1. Please ensure that your manuscript meets PLOS ONE's style requirements, including those for file naming. The PLOS ONE style templates can be found at

https://journals.plos.org/plosone/s/file?id=wjVg/PLOSOne_formatting_sample_main_body.pdf and

https://journals.plos.org/plosone/s/file?id=ba62/PLOSOne_formatting_sample_title_authors_affiliations.pdf

2. We note that you have stated that you will provide repository information for your data at acceptance. Should your manuscript be accepted for publication, we will hold it until you provide the relevant accession numbers or DOIs necessary to access your data. If you wish to make changes to your Data Availability statement, please describe these changes in your cover letter and we will update your Data Availability statement to reflect the information you provide.

3. We note that Figures 1 and 6 in your submission contain map images which may be copyrighted. All PLOS content is published under the Creative Commons Attribution License (CC BY 4.0), which means that the manuscript, images, and Supporting Information files will be freely available online, and any third party is permitted to access, download, copy, distribute, and use these materials in any way, even commercially, with proper attribution. For these reasons, we cannot publish previously copyrighted maps or satellite images created using proprietary data, such as Google software (Google Maps, Street View, and Earth). For more information, see our copyright guidelines: http://journals.plos.org/plosone/s/licenses-and-copyright.

We require you to either (1) present written permission from the copyright holder to publish these figures specifically under the CC BY 4.0 license, or (2) remove the figures from your submission:

3.1.    You may seek permission from the original copyright holder of Figures 1 and 6 to publish the content specifically under the CC BY 4.0 license. 

We recommend that you contact the original copyright holder with the Content Permission Form (http://journals.plos.org/plosone/s/file?id=7c09/content-permission-form.pdf) and the following text:

“I request permission for the open-access journal PLOS ONE to publish XXX under the Creative Commons Attribution License (CCAL) CC BY 4.0 (http://creativecommons.org/licenses/by/4.0/). Please be aware that this license allows unrestricted use and distribution, even commercially, by third parties. Please reply and provide explicit written permission to publish XXX under a CC BY license and complete the attached form.”

Please upload the completed Content Permission Form or other proof of granted permissions as an "Other" file with your submission.

In the figure caption of the copyrighted figure, please include the following text: “Reprinted from [ref] under a CC BY license, with permission from [name of publisher], original copyright [original copyright year].”

3.2.    If you are unable to obtain permission from the original copyright holder to publish these figures under the CC BY 4.0 license or if the copyright holder’s requirements are incompatible with the CC BY 4.0 license, please either i) remove the figure or ii) supply a replacement figure that complies with the CC BY 4.0 license. Please check copyright information on all replacement figures and update the figure caption with source information. If applicable, please specify in the figure caption text when a figure is similar but not identical to the original image and is therefore for illustrative purposes only.

The following resources for replacing copyrighted map figures may be helpful:

USGS National Map Viewer (public domain): http://viewer.nationalmap.gov/viewer/

The Gateway to Astronaut Photography of Earth (public domain): http://eol.jsc.nasa.gov/sseop/clickmap/

Maps at the CIA (public domain): https://www.cia.gov/library/publications/the-world-factbook/index.html and https://www.cia.gov/library/publications/cia-maps-publications/index.html

NASA Earth Observatory (public domain): http://earthobservatory.nasa.gov/

Landsat: http://landsat.visibleearth.nasa.gov/

USGS EROS (Earth Resources Observatory and Science (EROS) Center) (public domain): http://eros.usgs.gov/#

Natural Earth (public domain): http://www.naturalearthdata.com/

Reviewers' comments:

Reviewer #1: Review for PLOS ONE (PONE-D-20-405120)

Machado, A.F., Duarte Ritter, C., Lima Miranda, C., Ramos Pereira, M.J., Duarte, L., Potential mammalian species for investigating the past connections between Amazonia and the Atlantic forest.

This paper evaluates the geographic distribution of 127 mammalian species occurring in both Amazonia and the Atlantic rain forests. Potential connective routes in the past between both forested areas are identified. Three routes can be recognized; the present paper argues for a most important NE route whereas previous studies identified SE-NW route as the most frequently used.

Since the glacial forest refugia hypothesis of Haffer (Science 1969) a pleothora of studies have addressed the history of the extension of Amazonian rainforest. The history of Atlantic rain forest was often addressed in tandem. Due to the few available deep-time pollen records the dynamic history of Amazonian rainforest is still largely unknown (a ‘reasonable guess’ in the papers by M. Bush et al.) and still subject to much speculation. The history of the Atlantic rain forest is better known. In fact, Hoorn et al. (Science 2010) showed that the immense biodiversity originates from much older age as the Pleistocene, and that ice age cycles do not play an important role to explain high levels of biodiversity in Amazonia. This makes it interesting how old connecting routes should be. Are authors hinting to connecting routes of Pleistocene age?, perhaps even the most recent part of the Pleistocene? I am surprised that the Introduction does not place this study in a Pleistocene context providing arguments why a search for corridors could be potentially a contribution to this debate at large.

This study follows an interesting way of data analysis to recognize connecting routes between both forested areas. Basically I am surprised that the authors talk about ‘past connective routes’ (line 234) and do not a single effort to state more precisely ‘past’ along the time scale. However, with the history of Amazonian rain forest so poorly understood it is perhaps difficult to do a better job?

Although I am very much focused on botany, I follow the methods, but I cannot assess the methods used critically. Using Olson’s ecoregion polygons (line 152) is a reasonable approach.

As mentioned in the Discussion section (lines 210-211) this study reflects a new compilation of ever expanding data and results shows an incremental step forwards. Indeed, results may serve as a basis for future biogeographic studies considering different mammalian taxonomic groups (lines 98-99). As such this study is well designed, well presented and timely. I am not sure if the incremental step forwards is groundbreaking. Is this study able to bring more focus in the wide array of discussions about the history of Amazonian and Atlantic forest areas? The authors have not addressed this issue, making this fine study to a mammalian-specific palaeo-biogeographical contribution.

References: are not consistent (373. 377, 398)

Figure 1: make a better difference between the three colors of brown.

Figure 2: grey scale is unclear

Figures 1 and 5 could easily be combined.

In conclusion this is a fine study using an updated dataset and reaching relevant conclusions in a longstanding debate. This study is quite mammalian specific. I am wondering if the authors have tried to let results integrate in the palaeo-biogeographical discussion of Pleistocene Amazonian rainforest dynamics.

Reviewer #2: Review of Machado et al. Potential mammalian species for investigating the past connections between Amazonia 3 and the Atlantic Forest – PLOS ONE

Reviewer summary:

The authors assemble a list of potential mammal species that may help illuminate research into historic and pre-historic connection routes between the Amazon and the Atlantic Forest. Candidate species were identified as mammals with current distributions in both ecosystems. Potential dispersion routes were then identified by intersecting current ranges with ecoregions boundaries delimiting three potential pathways previously discussed in the literature on this topic. Finally, the candidate species list was queried against GenBank records to assess the quantity of available information by which to further explore the hypothesized routes.

Overall recommendation:

While this is a worthy exercise that may yield new insights into the paleo-ecological question, the paper as written does not present sufficient evidence to support the primary conclusion – that the NW pathway is most important – which runs contrary to the bulk of previous literature on the topic. The authors have assembled a nice baseline of information to support an intriguing new hypothesis, but major revisions to the framing of the paper would be required to cast this current work as a scientific finding.

Additional comments:

There is a substantial body of literature on this particular question – the authors would do well to more fully summarize the question at hand as well as existing scholarship so that readers not as familiar can better engage with their analysis. Although plenty of references are cited, a bit more set up is warranted.

The use of unmodified range polygons from IUCN is problematic for a precise vector-based analysis due to known uncertainties and omission/commission errors (see Brooks et al. 2019 https://doi.org/10.1016/j.tree.2019.06.009; Jung et al. 2020 https://doi.org/10.1038/s41597-020-00599-8)

Likewise, attribution of a particular dispersal route base on range intersection with precise ecoregion boundaries to me is not sufficient to assign a particular route at the exclusion of the other two – particularly when identifying dispersal routes through varying climate, land cover, ecoregion boundaries and species ranges back to 120,000 ybp. The authors, at a minimum, would need to examine the sensitivity of different ecoregion boundaries to delineate potential routes, discuss the paleo-ecology that may have variably facilitated the different routes and offer potential explanations/reasons that this evidence is sufficient to reconsider the literature as to the relative importance of routes.

The list of mammal species was queried against GenBank records, but there was no subsequent prioritization of species based on the quantity of sequences – would all be used? Just those with high data availability? The authors note: “However, it is important to highlight that, although many of these species have a large amount of available genetic data, it is common for many of the nucleotide sequences to contain missing information, which could render them unfit for use.” – it seems this next step would be necessary to include the GenBank availability in a species screening exercise.

The maps in figure 6 are difficult to reconcile vs. the known richness patterns of the Atlantic forest. For example, it appears as though in panel B – the majority of species ascribed to NE route only intersect the very northern portion of the Atlantic forest and there is not much richness of those species throughout the remainder of the Atlantic forest ecosystem. This pattern at least should be noted in the results/discussion. In panel D – only 1 species looks to have any range in the Atlantic forest – doesn’t that violate the first screening criterion?

There are minor grammatical and typographical errors throughout -- I am confident most/all would be caught in a revision.

The authors clearly have deeper insights and ideas for lines of inquiry into this question than are presented in this manuscript (as evidenced by some interesting but untested ideas in the discussion). As mentioned in the overall recommendation, both the objectives of this manuscript and the application of this dataset to future studies needs to be more precisely defined. I wish the authors all the best in their continued investigation into this question!

While revising your submission, please upload your figure files to the Preflight Analysis and Conversion Engine (PACE) digital diagnostic tool, https://pacev2.apexcovantage.com/. PACE helps ensure that figures meet PLOS requirements. To use PACE, you must first register as a user. Registration is free. Then, login and navigate to the UPLOAD tab, where you will find detailed instructions on how to use the tool. If you encounter any issues or have any questions when using PACE, please email PLOS at figures@plos.org. Please note that Supporting Information files do not need this step.

# Author comment: Author response to Decision Letter 0

**DOI:** 10.1371/journal.pone.0250016.r002

21 Feb 2021

We were happy to hear that you found our manuscript entitled: “Potential mammalian species for investigating the past connections between Amazonia and the Atlantic Forest” (PONE-D-20-40510) to be of interest. We found the comments and suggestions put forward during the review process to be most helpful and we thank you for the opportunity to submit this revised version of our work. 

Please see below for our responses to each point raised by the editor and the reviewers . Line numbers refer to the "clean" version of our manuscript (i.e. without track-changes).

On behalf of all other co-authors,

Camila D. Ritter. 

 

Point-by-point 

Editor’s requests:

1. “Please ensure that your manuscript meets PLOS ONE's style requirements, including those for file naming.” Reply/Action taken: We have checked the formatting style of PLOS ONE and formatted our manuscript according. We also made some additional changes to better meet PLOS ONE's requirements. For example, we chose to remove “Table 1” from the manuscript and keep it as supplementary material (“S1Table”), since keeping the table in the body of the text would have resulted in six pages and thus, an immense cut in the text. But we are open to revert this decision, in case the editor should prefer to keep S1 Table as part of the main article.

2. “We note that you have stated that you will provide repository information for your data at acceptance. Should your manuscript be accepted for publication, we will hold it until you provide the relevant accession numbers or DOIs necessary to access your data. If you wish to make changes to your Data Availability statement, please describe these changes in your cover letter and we will update your Data Availability statement to reflect the information you provide.” Reply/Action taken: We are sorry for the confusion. We meant to say that we used already freely available data from the repository databases, GenBank, IUCN, and Dinerstein et al. (2017) shapefiles. Thank you for drawing our attention to this mistake. 

3. “We note that Figures 1 and 6 in your submission contain map images which may be copyrighted. All PLOS content is published under the Creative Commons Attribution License (CC BY 4.0), which means that the manuscript, images, and Supporting Information files will be freely available online, and any third party is permitted to access, download, copy, distribute, and use these materials in any way, even commercially, with proper attribution. For these reasons, we cannot publish previously copyrighted maps or satellite images created using proprietary data, such as Google software (Google Maps, Street View, and Earth).” Reply/Action taken: We used shapefiles that are under CC BY 4.0 license. In Figure 4 (now Fig 5) we used animal silhouettes images from PHYLOPIC provided under CC BY 4.0 license. All citations and links are now provided in the figure legends.

While revising your submission, please upload your figure files to the Preflight Analysis and Conversion Engine (PACE) digital diagnostic tool, https://pacev2.apexcovantage.com/. PACE helps ensure that figures meet PLOS requirements. To use PACE, you must first register as a user. Registration is free. Then, login and navigate to the UPLOAD tab, where you will find detailed instructions on how to use the tool. If you encounter any issues or have any questions when using PACE, please email PLOS at figures@plos.org. Please note that Supporting Information files do not need this step. Reply/Action taken: We have uploaded our figures using PACE and checked that they meet the PLOS requirements.

 

Reviewer #1:

1. “Since the glacial forest refugia hypothesis of Haffer (Science 1969) a pleothora of studies have addressed the history of the extension of Amazonian rainforest. The history of Atlantic rain forest was often addressed in tandem. Due to the few available deep-time pollen records the dynamic history of Amazonian rainforest is still largely unknown (a ‘reasonable guess’ in the papers by M. Bush et al.) and still subject to much speculation. The history of the Atlantic rain forest is better known. In fact, Hoorn et al. (Science 2010) showed that the immense biodiversity originates from much older age as the Pleistocene, and that ice age cycles do not play an important role to explain high levels of biodiversity in Amazonia. This makes it interesting how old connecting routes should be. Are authors hinting to connecting routes of Pleistocene age? Perhaps even the most recent part of the Pleistocene? I am surprised that the Introduction does not place this study in a Pleistocene context providing arguments why a search for corridors could be potentially a contribution to this debate at large.” Reply/Action taken: We agree regarding the importance of anchoring this study in time. We have therefore reviewed the introduction to include more information on the origins of the tropical rainforests in South America and their changes over time, which are important for understanding the connections between Amazonia and the Atlantic forest. The introduction now reads: “The origin of the tropical rainforests in South America is dated to at least 65 million years ago (mya) [15-17]. Since then, these forests have undergone several changes, expanding and retracting. From the Oligocene (~ 23 mya) to the Pliocene (~ 3 mya), successive tectonic events led to the Andean uplift, restricting the entry of rainfall from the Pacific into the interior of the continent resulting in a drier climate with a forest reduction and the expansion of savannas, giving rise to the dry diagonal, and, consequently, the separation of Amazonia and the Atlantic Forest [15, 18-20]. Furthermore, there is evidence of more recent forest expansions and retractions caused by Quaternary climatic fluctuations, such as glacial cycles during the Pleistocene and recurrent periods of extreme rainfall during the Holocene [11-14]. Indeed, the high rates of vegetation cover changes in the dry diagonal and the Atlantic Forest [21, 22] suggest not just ancient but also recent connections and disruptions between Amazonia and the Atlantic Forest [9]” (lines 50-61).

2. “This study follows an interesting way of data analysis to recognize connecting routes between both forested areas. Basically I am surprised that the authors talk about ‘past connective routes’ (line 234) and do not a single effort to state more precisely ‘past’ along the time scale. However, with the history of Amazonian rain forest so poorly understood it is perhaps difficult to do a better job?” Reply/Action taken: The reviewer correctly observes that it is a difficult job to anchor the connective routes between Amazonia and the Atlantic forest in time, but by creating a list over mammalian species that contains geographical, ecological, and genetic information we present a first and important step in this direction. To elucidate the history of the past routes and precisely place them along the timescale would however require phylogenetic and phylogeographic analyses that are beyond the scope of this paper. Yet, we agree that this will be key for understanding the evolution and dynamics of Amazonia and the Atlantic Forest. Therefore, in the introduction, as shown above, we have made an effort to better elaborate on what is known about the time scale of these connections, as well as possible sample biases. 

In addition, we added the following to the discussion: “Because most data previously used to test the existence of the connective routes over time came from interspecific molecular evidence from a few species, and because most species split before the Plio-Pleistocene (~5 mya) [60], such data may have biased results toward older routes, e.g., toward the Western route which would have occurred for the first time at least between ~30 to 20 mya in the Oligo-Miocene [5, 8, 10]. In contrast, studies on paleo vegetation, pollen data and biogeographic approaches indicate that several past connections through the Eastern route could have occurred during the Pleistocene (~2.5 mya) [11-14, 21, 29, 58-59]. Hence, these insights highlight a need of more intraspecific studies that look at the genetic divergence within multiple taxa since such studies may shed light on the complexity of the evolution and existence of the connections between Amazonia and the Atlantic Forest over time [76-79].” (lines 297-307). 

In the discussion we also state clearly that our objective with this study and the species list is to: “subsidize future mammal phylogenetic and phylogeographic studies to shed light on the temporal and spatial use of the connections between Amazonia and the Atlantic Forest in relation to the ecology and evolution of South American mammals” (lines 290-292).

3. “Although I am very much focused on botany, I follow the methods, but I cannot assess the methods used critically. Using Olson’s ecoregion polygons (line 152) is a reasonable approach.” Reply/Action taken: We used Olson’s ecoregion polygons to delimit the extent of the routes. This ecoregion database is the most complete database for ecoregions of the world [45]. Furthermore, our route regions agree with the previous literature [5, 6, 23, 25].

4. “As mentioned in the Discussion section (lines 210-211) this study reflects a new compilation of ever expanding data and results shows an incremental step forwards. Indeed, results may serve as a basis for future biogeographic studies considering different mammalian taxonomic groups (lines 98-99). As such this study is well designed, well presented and timely. I am not sure if the incremental step forwards is groundbreaking. Is this study able to bring more focus in the wide array of discussions about the history of Amazonian and Atlantic forest areas? The authors have not addressed this issue, making this fine study to a mammalian-specific palaeo-biogeographical contribution.” Reply/Action taken: Building on our data compilation future studies will be able to bring new insights that contribute to the wide array of discussions about the history of Amazonian and Atlantic forest areas, for example through phylogenetic and phylogeographic analysis that are beyond the scope of this study. To compile mammalian geographical, ecological, and genetic data is difficult and time consuming, therefore we decided to make this list available to assist researchers interested in South American mammal biogeography. We appreciate this reviewer's comment, and we restructured the discussion to be clearer and more direct about our findings.

5. “References: are not consistent (373. 377, 398)” Reply/Action taken: We have checked all references to ensure consistency in this new version.

6. “Figure 1: make a better difference between the three colors of brown.” Reply/Action taken: We thank the reviewer for the suggestion. We now use higher contrast colours and we have checked all figures for colour-blind mode.

7. “Figure 2: grey scale is unclear.” Reply/Action taken: We now use a more contrasting grey scale, and this figure is now the Fig 3 to better structure the order of mention of the results.

8. “Figures 1 and 5 could easily be combined.” Reply/Action taken: We thank the reviewer for the suggestion. We merged figures 1 and 5.

9. “In conclusion this is a fine study using an updated dataset and reaching relevant conclusions in a longstanding debate. This study is quite mammalian specific. I am wondering if the authors have tried to let results integrate in the palaeo-biogeographical discussion of Pleistocene Amazonian rainforest dynamics.” Reply/Action taken: We now contextualize the time scale of the proposed routes in both the introduction and the discussion by integrating information from palaeo-biogeographical studies. However, we do not directly discuss the Pleistocene Amazonian rainforest dynamics in depth because it is not the objective of the study and because the Pleistocene cycles appear to have been less pronounced inside Amazonia [20]. Indeed, the environmental stability is proposed to have much been greater in Amazonia throughout the late Quaternary, whereas there were high rates of changes in vegetation cover in the dry diagonal and the Atlantic Forest [21, 22]. We do however elaborate on the impacts of the Pleistocene dynamics on forest expansions and retractions in the dry diagonal leading to the establishment of connection routes between Amazonia and the Atlantic Forest.

 

Reviewer #2:

Overall recommendation:

1. “While this is a worthy exercise that may yield new insights into the paleo-ecological question, the paper as written does not present sufficient evidence to support the primary conclusion – that the NW pathway is most important – which runs contrary to the bulk of previous literature on the topic. The authors have assembled a nice baseline of information to support an intriguing new hypothesis, but major revisions to the framing of the paper would be required to cast this current work as a scientific finding.” Reply/Action taken: We agree and therefore reviewed the text to strengthen our findings with arguments in accordance with the literature. We highlight that the limited number of species used in previous studies could have biased their conclusions but also stress that further studies are necessary to test this proposition and our hypothesis about the importance of the route which we refer to as the Eastern route. We thus hope to clarify that also we call for further studies, which are necessary if we are to shed light on the past connections between Amazonia and the Atlantic Forest. It is for this reason that we make our mammal list with ecological, geographical and genetic data widely available.

Additional comments:

2. “There is a substantial body of literature on this particular question – the authors would do well to more fully summarize the question at hand as well as existing scholarship so that readers not as familiar can better engage with their analysis. Although plenty of references are cited, a bit more set up is warranted. The use of unmodified range polygons from IUCN is problematic for a precise vector-based analysis due to known uncertainties and omission/commission errors (see Brooks et al. 2019 https://doi.org/10.1016/j.tree.2019.06.009; Jung et al. 2020 https://doi.org/10.1038/s41597-020-00599-8).” Reply/Action taken: We understand the concern in relation to the IUCN expert maps, since there is great disbelief in this data for other taxonomic groups. However, these maps are created and verified by specialists based on occurrence records, with the predicted presence being restricted to areas with adequate habitat where the species is known and adhering to the precautionary principle that guides conservation efforts. Although these maps were designed for conservation purposes, they are widely used. In addition, they have been extensively updated to consider taxonomic revisions and fragmentation of mammal distributions. Using occurrence maps generated by species-level experts is a better option for larger-scale studies than using occurrence records, due to the extensive errors associated with the latter, such as problems with false positives due to erroneous taxonomic identifications and errors in coordinates. Finally, studies testing the use of occurrence records versus IUCN maps have shown that specialized knowledge provides more robust predictions and/or correlated results. Thus, we justify our use of the IUCN data in the following way: “We used the IUCN distribution data since these maps are created and verified by specialists based on occurrence records already checked and thus restrict species occurrences to areas with presumably suitable habitat where the species is known, following a precautionary principle to guide conservation efforts [35, 36]. Although these maps were designed for conservation purposes and recent studies suggest new methods for improving the IUCN maps and classifications [37, 38], these maps have proved to be an important source of information for many macroecological studies [39-44] and represent the most complete currently available species distribution maps for different mammal taxon”. (lines 118-125).

3. “Likewise, attribution of a particular dispersal route base on range intersection with precise ecoregion boundaries to me is not sufficient to assign a particular route at the exclusion of the other two – particularly when identifying dispersal routes through varying climate, land cover, ecoregion boundaries and species ranges back to 120,000 ybp. The authors, at a minimum, would need to examine the sensitivity of different ecoregion boundaries to delineate potential routes, discuss the paleo-ecology that may have variably facilitated the different routes and offer potential explanations/reasons that this evidence is sufficient to reconsider the literature as to the relative importance of routes.” Reply/Action taken: We consider the regions previously described in the literature. These studies cover not only molecular data, but also geological, climatic and palaeoecological data. The ecoregions' shapefile was used as an initial base for the delimitation of the routes, but the final result of the delimited area being a mixture of information from areas previously described in the literature based on molecular data, geological, climatic and palaeoecological data complemented ecoregion data. Now, figure 1 includes the areas delimited by us for each route. Although we consider these areas an adequate starting point for investigating the past connections between Amazonia and the Atlantic forest, further studies are needed to confirm our results. Throughout the discussion, we therefore highlight that this initial compilation is to serve as basis for future studies.

4. “The list of mammal species was queried against GenBank records, but there was no subsequent prioritization of species based on the quantity of sequences – would all be used? Just those with high data availability? The authors note: “However, it is important to highlight that, although many of these species have a large amount of available genetic data, it is common for many of the nucleotide sequences to contain missing information, which could render them unfit for use.” – it seems this next step would be necessary to include the GenBank availability in a species screening exercise.” Reply/Action taken: Yes, further analysis and curation of the genetic mammal data are necessary to investigate the past connections between Amazonia and the Atlantic forest. Next steps would include GenBank sequences curation. This would require quite some effort for the 127 species (more than 150,000 sequences in total) since, unfortunately, Genbank does not provide any location-based data filtering tools (which would be important for future phylogeographic studies). In this study our goal was simply to provide a list of mammalian species with a potential for investigating the historic South Americas forest dynamics and their ecologic, geographic and genetic data. From our own experience, this list was essential to select species to be used in our research. Therefore, we want to share these data to facilitate for future phylogeographic studies. However, the inclusion of species in such future studies, would depend on their exact research questions.

5. “The maps in figure 6 are difficult to reconcile vs. the known richness patterns of the Atlantic forest. For example, it appears as though in panel B – the majority of species ascribed to NE route only intersect the very northern portion of the Atlantic forest and there is not much richness of those species throughout the remainder of the Atlantic forest ecosystem. This pattern at least should be noted in the results/discussion. In panel D – only 1 species looks to have any range in the Atlantic forest – doesn’t that violate the first screening criterion?.” Reply/Action taken: We appreciate the concern expressed by the reviewer and acknowledge that the previous figure may not have made our data justice. We therefore reworked the figure to include all species distribution patterns (i.e., also species that evidenced use of multiple routes). Including all species led us to identify an error in the tabulation of the data (i.e., an erroneous combination of routes), which we have now corrected for. However, these changes have not changed the main results (i.e., the pattern found has remained the same). This correction resulted in an increase in the number of species that evidence past use of the Eastern route, and with that, the results now show more species that occur throughout the entire Atlantic Forest, which can be seen in the new figure 2 (we changed the order of the figures to better structure the results). As for the species in panel D, all 3 species that occur on that route reach the limits of the Atlantic Forest. Due to little overlap in the distributions of these species, it is apparent that only one of them reaches into the interior of the Atlantic Forest. Two of them occur in this biome in larger areas (one occurring up to the South of the Atlantic Forest and another to the Central Atlantic Forest) and one of them occur at the edge of the Atlantic Forest (in the forests of the Paraná). Considering that there are forests that extend into the ecotone between the Atlantic Forest and the other biomes (for example, extensions of humid and seasonally humid forests in the north and northeast of Argentina) it would be prudent not to exclude this species from the list.

6. “There are minor grammatical and typographical errors throughout -- I am confident most/all would be caught in a revision.” Reply/Action taken: We apologize for these mistakes and carefully checked the text to avoid any errors in this new version.

7. “The authors clearly have deeper insights and ideas for lines of inquiry into this question than are presented in this manuscript (as evidenced by some interesting but untested ideas in the discussion). As mentioned in the overall recommendation, both the objectives of this manuscript and the application of this dataset to future studies needs to be more precisely defined. I wish the authors all the best in their continued investigation into this question!” Reply/Action taken: We thank the reviewer for this comment. Indeed, we created this species list to select potential mammal species for investigating the connections between Amazonia and the Atlantic Forest in other manuscripts. As collecting and filtering all data was hard and time consuming, we decided to share this list as an open access manuscript to help other researchers interested in using mammals as study objects in phylogeographic and phylogenetic studies to investigate past dispersal and forest dynamics.

Attachment Submitted filename: Response_to_Reviewers.docx Click here for additional data file.

# Peer review: Decision Letter 1

**DOI:** 10.1371/journal.pone.0250016.r003

**Authors:** Paulo Corti

11 Mar 2021

PONE-D-20-40510R1

Potential mammalian species for investigating the past connections between Amazonia and the Atlantic Forest

PLOS ONE

Dear Dr. ritter,

Thank you for submitting your manuscript to PLOS ONE. After careful consideration, we feel that it has merit but does not fully meet PLOS ONE’s publication criteria as it currently stands. Therefore, we invite you to submit a revised version of the manuscript that addresses the points raised during the review process.

Pay special attention to reviewer #2 comments in relation to the support for your conclusions reviewer is asking. I agree with its argument that you should provide stronger evidence for your primary conclusions. You mention some warnings about your results in the discussion, but this is not kept in the abstract.

Please submit your revised manuscript by Apr 25 2021 11:59PM. If you will need more time than this to complete your revisions, please reply to this message or contact the journal office at plosone@plos.org. When you're ready to submit your revision, log on to https://www.editorialmanager.com/pone/ and select the 'Submissions Needing Revision' folder to locate your manuscript file.

Please include the following items when submitting your revised manuscript:

- A rebuttal letter that responds to each point raised by the academic editor and reviewer(s). You should upload this letter as a separate file labeled 'Response to Reviewers'.
- A marked-up copy of your manuscript that highlights changes made to the original version. You should upload this as a separate file labeled 'Revised Manuscript with Track Changes'.
- An unmarked version of your revised paper without tracked changes. You should upload this as a separate file labeled 'Manuscript'.

If you would like to make changes to your financial disclosure, please include your updated statement in your cover letter. Guidelines for resubmitting your figure files are available below the reviewer comments at the end of this letter.

If applicable, we recommend that you deposit your laboratory protocols in protocols.io to enhance the reproducibility of your results. Protocols.io assigns your protocol its own identifier (DOI) so that it can be cited independently in the future. For instructions see: http://journals.plos.org/plosone/s/submission-guidelines#loc-laboratory-protocols

We look forward to receiving your revised manuscript.

Kind regards,

Paulo Corti, Ph.D.

Academic Editor

PLOS ONE

Please review your reference list to ensure that it is complete and correct. If you have cited papers that have been retracted, please include the rationale for doing so in the manuscript text, or remove these references and replace them with relevant current references. Any changes to the reference list should be mentioned in the rebuttal letter that accompanies your revised manuscript. If you need to cite a retracted article, indicate the article’s retracted status in the References list and also include a citation and full reference for the retraction notice.

[Note: HTML markup is below. Please do not edit.]

**Comments to the Author**

Please use the space provided to explain your answers to the questions above. You may also include additional comments for the author, including concerns about dual publication, research ethics, or publication ethics. (Please upload your review as an attachment if it exceeds 20,000 characters)

Reviewer #1: I am fine with the improvements of the manuscript and the explanation.

Note: I found the reference of Hoorn et al. (2010) with an incomplete list of authors (line 454).

Reviewer #2: I thank the authors for their attention to my previous comments and appreciate their responses. However, I still feel that the primary conclusion of the manuscript – that the Eastern route is/was most commonly used – is not supported by the evidence presented. The authors do well to caveat their result in the discussion, but the conclusion remains plainly stated in the abstract (separately it appears the previous nomenclature for the dispersal routes remains in the abstract – i.e. ‘northeastern’ vs. ‘Eastern’).

What the authors have found is that the present-day geography of the mammal species found in both the Amazon and the Atlantic Forests points to more species in the northeastern portion of the dry diagonal (and adjoining forested habitats). It remains to be seen if this present-day geography can offer any conclusions as to the paleo dispersal routes. The authors state in their response “Throughout the discussion, we therefore highlight that this initial compilation is to serve as basis for future studies.” I am recommending minor revisions to properly contextualize the conclusion of the most commonly used dispersal routes as a preliminary geography-based finding throughout – but especially in the abstract.

I find the authors’ response to the question of the Genbank records as a means to further prioritize this list to be unsatisfactory. If the intent of this list is to guide future investigation into this question of dispersal route, wouldn’t it make sense to hear from the authors as to what level of genetic information is sufficient or at least more preferable? Indeed, it depends on the research question, but surely the authors have recommendations for species among the 127 assessed as to which are more ripe for immediate future study and/or if there are species that could be strategically targeted for additional sampling to help confirm/deny a particular route.

The maps in Fig 6 are much improved – and I thank the authors for their explanation (which in retrospect makes perfect sense). Thinking further, I am struck by how continuous the distributions of species attributed to the Eastern route are across the dry diagonal between the forest habitat – albeit biased toward the coast. This is actually a rather compelling argument that the Eastern route may have been preferred (possible access to coastal habitats en route)!

My best wishes to the authors

**********

7. PLOS authors have the option to publish the peer review history of their article (what does this mean?). If published, this will include your full peer review and any attached files.

If you choose “no”, your identity will remain anonymous but your review may still be made public.

**Do you want your identity to be public for this peer review?** For information about this choice, including consent withdrawal, please see our Privacy Policy.

Reviewer #1: **Yes: **Henry Hooghiemstra

Reviewer #2: No

[NOTE: If reviewer comments were submitted as an attachment file, they will be attached to this email and accessible via the submission site. Please log into your account, locate the manuscript record, and check for the action link "View Attachments". If this link does not appear, there are no attachment files.]

While revising your submission, please upload your figure files to the Preflight Analysis and Conversion Engine (PACE) digital diagnostic tool, https://pacev2.apexcovantage.com/. PACE helps ensure that figures meet PLOS requirements. To use PACE, you must first register as a user. Registration is free. Then, login and navigate to the UPLOAD tab, where you will find detailed instructions on how to use the tool. If you encounter any issues or have any questions when using PACE, please email PLOS at figures@plos.org. Please note that Supporting Information files do not need this step.

# Author comment: Author response to Decision Letter 1

**DOI:** 10.1371/journal.pone.0250016.r004

18 Mar 2021

Point-by-point 

Reviewer #1:

1. “I am fine with the improvements of the manuscript and the explanation.

Note: I found the reference of Hoorn et al. (2010) with an incomplete list of authors (line 454).”

Author’s response:

Thank you for your attention. We corrected and standardized all references so that references with more than six authors read “et al.” from the sixth author on – thus, following the journal’s online publication guideline.

 

Reviewer #2:

2. “I thank the authors for their attention to my previous comments and appreciate their responses. However, I still feel that the primary conclusion of the manuscript – that the Eastern route is/was most commonly used – is not supported by the evidence presented. The authors do well to caveat their result in the discussion, but the conclusion remains plainly stated in the abstract (separately it appears the previous nomenclature for the dispersal routes remains in the abstract – i.e. ‘northeastern’ vs. ‘Eastern’). What the authors have found is that the present-day geography of the mammal species found in both the Amazon and the Atlantic Forests points to more species in the northeastern portion of the dry diagonal (and adjoining forested habitats). It remains to be seen if this present-day geography can offer any conclusions as to the paleo dispersal routes. The authors state in their response “Throughout the discussion, we therefore highlight that this initial compilation is to serve as basis for future studies.” I am recommending minor revisions to properly contextualize the conclusion of the most commonly used dispersal routes as a preliminary geography-based finding throughout – but especially in the abstract.”

Author’s response: 

Thank you for these suggestions. We edited parts of the abstract (lines 29-30 and 37-41) to better clarify the study's findings and the limits of the interpretations. We also edited some excerpts throughout the text to strengthen our findings (lines 291, 300, 302-306, 320-323, 355, 378-379, 381-390, 396, and 400-405). Finally, we changed the names of the routes in the abstract (line 26) to standardize the nomenclature throughout the text.

3. “I find the authors’ response to the question of the Genbank records as a means to further prioritize this list to be unsatisfactory. If the intent of this list is to guide future investigation into this question of dispersal route, wouldn’t it make sense to hear from the authors as to what level of genetic information is sufficient or at least more preferable? Indeed, it depends on the research question, but surely the authors have recommendations for species among the 127 assessed as to which are more ripe for immediate future study and/or if there are species that could be strategically targeted for additional sampling to help confirm/deny a particular route.”

Author’s response: 

We appreciate the reviewer comment and now specify that “Depending on the geographic distribution of data, species with regular to high availability of genetic data could serve” for investigating the past connection routes (lines 379-380). To exemplify this we give details about three species that could either be used immediately or after strategic sampling, to investigate the Eastern and Central routes, see lines 383-391: “For instance, Marmosa demerarae and Marmosa murina had regular (n = 47 sequences) and intermediate (n = 77 sequences) genetic availability, but adequate geographic distribution of samples (totalling 31 and 39 localities along their respective ranges) and hence serve for investigating the Eastern and Central routes respectively (Machado et al., in prep.). An example of a species with low genetic availability (n = 13 sequences) but good geographic coverage (11 localities) was Caluromys philander. Increasing the number of sequences per locality would thus render C. philander a good candidate for investigating the Central route. For other species increasing the geographical coverage rather than the number of sequences per locality may be more important”. Additionally, we have included both text and Fig 5 results for availability of genetic data by order, from lines 277-279: "The availability of genetic data was mostly high and intermediate for most orders. Some orders had regular data and few orders had low availability of genetic data". We also edited the count for categorized species with low availability of genetic data, so that these vary between one and 22 sequences (line 160), thus removing species without genetic data from this category. Species without genetic data are listed in the supplementary material with “absent” genetic availability (S1 Table), and consequently, we edited Fig. 4 to reflect this exclusion of species with “absent” genetic availability. You may also note that we changed the names of the categories to “low”, “regular”, “intermediate” and “high”, since we felt that these were more adequate.

4. “The maps in Fig 6 are much improved – and I thank the authors for their explanation (which in retrospect makes perfect sense). Thinking further, I am struck by how continuous the distributions of species attributed to the Eastern route are across the dry diagonal between the forest habitat – albeit biased toward the coast. This is actually a rather compelling argument that the Eastern route may have been preferred (possible access to coastal habitats en route)!

My best wishes to the authors”

Author’s response: 

We thank the reviewer for his/her comment. We added the following to the discussion (lines 303-307): “These species [attributed to the Eastern route] also evidenced strikingly continuous distributions across the dry diagonal between the forest habitats – albeit biased toward the coast. Thus, their present-day geography provides a compelling indication that the Eastern route, with its possible access to coastal habitats, may have been preferred over the other routes”.

Attachment Submitted filename: Response_to_Reviewers_R2_final.docx Click here for additional data file.

# Peer review: Decision Letter 2

**DOI:** 10.1371/journal.pone.0250016.r005

**Authors:** Paulo Corti

30 Mar 2021

Potential mammalian species for investigating the past connections between Amazonia and the Atlantic Forest

PONE-D-20-40510R2

Dear Dr. ritter,

We’re pleased to inform you that your manuscript has been judged scientifically suitable for publication and will be formally accepted for publication once it meets all outstanding technical requirements.

Within one week, you’ll receive an e-mail detailing the required amendments. When these have been addressed, you’ll receive a formal acceptance letter and your manuscript will be scheduled for publication.

An invoice for payment will follow shortly after the formal acceptance. To ensure an efficient process, please log into Editorial Manager at http://www.editorialmanager.com/pone/, click the 'Update My Information' link at the top of the page, and double check that your user information is up-to-date. If you have any billing related questions, please contact our Author Billing department directly at authorbilling@plos.org.

If your institution or institutions have a press office, please notify them about your upcoming paper to help maximize its impact. If they’ll be preparing press materials, please inform our press team as soon as possible -- no later than 48 hours after receiving the formal acceptance. Your manuscript will remain under strict press embargo until 2 pm Eastern Time on the date of publication. For more information, please contact onepress@plos.org.

Kind regards,

Paulo Corti, Ph.D.

Academic Editor

PLOS ONE

**Comments to the Author**

1. If the authors have adequately addressed your comments raised in a previous round of review and you feel that this manuscript is now acceptable for publication, you may indicate that here to bypass the “Comments to the Author” section, enter your conflict of interest statement in the “Confidential to Editor” section, and submit your "Accept" recommendation.

Reviewer #2: All comments have been addressed

**********

2. Is the manuscript technically sound, and do the data support the conclusions?

The manuscript must describe a technically sound piece of scientific research with data that supports the conclusions. Experiments must have been conducted rigorously, with appropriate controls, replication, and sample sizes. The conclusions must be drawn appropriately based on the data presented. 

Reviewer #2: Yes

**********

3. Has the statistical analysis been performed appropriately and rigorously? 

Reviewer #2: Yes

**********

4. Have the authors made all data underlying the findings in their manuscript fully available?

The PLOS Data policy requires authors to make all data underlying the findings described in their manuscript fully available without restriction, with rare exception (please refer to the Data Availability Statement in the manuscript PDF file). The data should be provided as part of the manuscript or its supporting information, or deposited to a public repository. For example, in addition to summary statistics, the data points behind means, medians and variance measures should be available. If there are restrictions on publicly sharing data—e.g. participant privacy or use of data from a third party—those must be specified.

Reviewer #2: Yes

**********

5. Is the manuscript presented in an intelligible fashion and written in standard English?

PLOS ONE does not copyedit accepted manuscripts, so the language in submitted articles must be clear, correct, and unambiguous. Any typographical or grammatical errors should be corrected at revision, so please note any specific errors here.

Reviewer #2: Yes

**********

6. Review Comments to the Author

Please use the space provided to explain your answers to the questions above. You may also include additional comments for the author, including concerns about dual publication, research ethics, or publication ethics. (Please upload your review as an attachment if it exceeds 20,000 characters)

Reviewer #2: I thank the authors for their prompt (!) consideration of my feedback. In my opinion the manuscript is much improved -- and makes a clear case for re-examination of paleo-dispersal routes with a roadmap to do so. Please note I have read for content and have not done a thorough proof-read for typographical/formatting errors.

My best

# Editor report: Acceptance letter

**DOI:** 10.1371/journal.pone.0250016.r006

**Authors:** Paulo Corti

1 Apr 2021

PONE-D-20-40510R2 

Potential mammalian species for investigating the past connections between Amazonia and the Atlantic Forest 

Dear Dr. Ritter:

I'm pleased to inform you that your manuscript has been deemed suitable for publication in PLOS ONE. Congratulations! Your manuscript is now with our production department. 

If your institution or institutions have a press office, please let them know about your upcoming paper now to help maximize its impact. If they'll be preparing press materials, please inform our press team within the next 48 hours. Your manuscript will remain under strict press embargo until 2 pm Eastern Time on the date of publication. For more information please contact onepress@plos.org.

If we can help with anything else, please email us at plosone@plos.org. 

Thank you for submitting your work to PLOS ONE and supporting open access. 

Kind regards, 

PLOS ONE Editorial Office Staff

on behalf of

Dr. Paulo Corti 

Academic Editor

PLOS ONE
